# Differential Expression of Immune Genes between Two Closely Related Beetle Species with Different Immunocompetence following Attack by *Asecodes parviclava*

**DOI:** 10.1093/gbe/evaa075

**Published:** 2020-04-13

**Authors:** Xuyue Yang, Lisa Fors, Tanja Slotte, Ulrich Theopold, Mahesh Binzer-Panchal, Christopher W Wheat, Peter A Hambäck

**Affiliations:** e1 Department of Ecology, Environment and Plant Sciences, Stockholm University, Sweden; e2 Department of Molecular Biosciences, The Wenner-Gren Institute, Stockholm University, Sweden; e3 Department of Medical Biochemistry and Microbiology, National Bioinformatics Infrastructure Sweden (NBIS), Science for Life Laboratory, Uppsala University, Sweden; e4 Department of Zoology, Stockholm University, Sweden

**Keywords:** parasitoid wasp, *Galerucella*, transcriptome, insect immunity, hematopoiesis

## Abstract

Endoparasitoid wasps are important natural enemies of many insect species and are major selective forces on the host immune system. Despite increased interest in insect antiparasitoid immunity, there is sparse information on the evolutionary dynamics of biological pathways and gene regulation involved in host immune defense outside *Drosophila* species. We de novo assembled transcriptomes from two beetle species and used time-course differential expression analysis to investigate gene expression differences in closely related species *Galerucella pusilla* and *G. calmariensis* that are, respectively, resistant and susceptible against parasitoid infection by *Asecodes parviclava* parasitoids. Approximately 271 million and 224 million paired-ended reads were assembled and filtered to form 52,563 and 59,781 transcripts for *G. pusilla* and *G. calmariensis*, respectively. In the whole-transcriptome level, an enrichment of functional categories related to energy production, biosynthetic process, and metabolic process was exhibited in both species. The main difference between species appears to be immune response and wound healing process mounted by *G. pusilla* larvae. Using reciprocal BLAST against the *Drosophila melanogaster* proteome, 120 and 121 immune-related genes were identified in *G. pusilla* and *G. calmariensis*, respectively. More immune genes were differentially expressed in *G. pusilla* than in *G. calmariensis*, in particular genes involved in signaling, hematopoiesis, and melanization. In contrast, only one gene was differentially expressed in *G. calmariensis*. Our study characterizes important genes and pathways involved in different immune functions after parasitoid infection and supports the role of signaling and hematopoiesis genes as key players in host immunity in *Galerucella* against parasitoid wasps.

## Introduction

Parasitoid attack is a major cause of mortality in many insect species (Godfray and Shimada 1999; Asgari and Rivers 2011), exerting strong selection on traits that affect the likelihood or the outcome of an attack. Defense mechanisms against endoparasitoids that complete their development inside the host often involve the host immune system, which evolves quickly in response to the changing virulence of endoparasitoids and shows large variation among species (Carton et al. 2008). For instance, *Drosophila* species show great variation in immunocompetence against the parasitoid *Asobara tabida*, where most species in the *Drosophila obscura* group show immune deficiency and no egg encapsulation, whereas other taxa show very strong immune responses (Eslin and Doury 2006; Havard et al. 2009; Singh et al. 2009; Salazar-Jaramillo et al. 2014). Similar patterns have recently been documented in the leaf beetle genus *Galerucella*, where three closely related species sharing a common parasitoid wasp enemy differ regarding parasitism rate, encapsulation success, and hemocyte production (Fors et al. 2014, 2016).

The cellular basis underlying variation in immunocompetence against endoparasitoids in nonmodel insects such as *Galerucella* is largely similar to *Drosophila* (Fors et al. 2014). In general, the immune response following a parasitoid attack starts with the recognition of the parasitoid egg. Immune signals subsequently induce a rapid increase and differentiation of hemocytes that attach to the egg surface, leading to the formation of a capsule around the egg with multiple layers of hemocytes (Carton et al. 2008). In *Drosophila*, this encapsulation process involves three major hemocyte types: plasmatocytes, lamellocytes, and crystal cells. Plasmatocytes are mainly responsible for phagocytosis and make up the majority of circulating hemocytes (>95%). The latter also consists of a small proportion of crystal cells (<5%), which contain crystalline enzymes that are required for humoral melanization (Schmid et al. 2019). Finally, lamellocytes are specifically produced after attack by parasitoids and participate in capsule formation (Lavine and Strand 2002; Meister and Lagueux 2003). Encapsulation often ends with melanization, which is the release and activation of phenoloxidase, leading to blackening at the wound site or around the encapsulated egg (Honti et al. 2014). Parasitoid eggs inside the capsule normally die from asphyxiation within 48 h, under the joint effect of encapsulation and melanization (Wertheim et al. 2005).

In *Galerucella*, six types of hemocytes have been discovered based on their morphologies, and three (lamellocytes, phagocytes, and granulocytes) were specifically involved in the encapsulation process (Fors et al. 2014). Lamellocytes, which serve the same function as in *Drosophila melanogaster*, are essential for capsule formation to be completed. Phagocytes, although not the major factor, also contribute to the encapsulation process in *Galerucella* (Carton et al. 2008; Fors et al. 2014). Finally, granulocytes in *Galerucella* share their mode of secretion with crystal cells in *Drosophila* (Fors et al. 2014), which are involved in wound and capsule melanization.

Although the hemocytes underlying insect immune responses are largely characterized, their regulation is only partly understood and primarily characterized in *Drosophila*. The genes involved in immune responses can be classified in seven functional categories, as proposed by Salazar-Jaramillo et al. (2014). The first class of genes involves recognition genes, for instance, peptidoglycan recognition proteins (PGRPs) and Gram-negative bacteria-binding proteins that are involved in the recognition of foreign objects, such as parasitoid eggs. The second class of genes involves signaling genes for transduction pathways (Toll, JAK-STAT, JNK, and IMD) that initiate the proliferation of hemocytes needed to encapsulate the foreign object. The third class involves effector genes coding for antimicrobial peptides that target bacterial or fungal membranes, leading to damage of the membrane and death of the cell (Bulet et al. 1999). The fourth class of genes involves modulating genes coding for serpins and serine proteases with mostly unknown immune function (e.g., *Spn88Eb*). The fifth class comprises hematopoiesis genes that are specifically involved in parasitoid-related immunity (hemocyte differentiation, proliferation, and regulation of hematopoiesis process, e.g., *gcm*, *wg*, and *yantar*). The sixth category includes genes coding for prophenoloxidases and their regulators (e.g., *PPO3*, *yellow*), which produce cytotoxic as well as crosslinking intermediates and ultimately melanin needed to complete the capsule around the parasitoid egg. The final class of genes is mainly those coding for chitinases responsible for the healing of wounds after parasitoid oviposition. The elucidation of these genes and pathways has significantly facilitated our understanding of the evolutionary dynamics acting upon genes involved in antiparasitoid immunity, but their occurrence and relevance outside *Drosophila* species have received little attention.

A powerful approach for understanding differences in immune responses is to compare gene expression patterns between closely related species that differ in their immunocompetence. Here, we examined differences in gene expression following parasitoid attack in the closely related beetles *Galerucella pusilla* and *G. calmariensis* (Coleoptera: Chrysomelidae). They share the same host plant, *Lythrum salicaria* (Lythraceae) and are attacked by the same koinobiont larval parasitoid, *Asecodes parviclava* (Hymenoptera: Eulophidae) (Fors et al. 2014). These species were selected for study as they exhibit large differences in their capacity to encapsulate parasitoid eggs, despite having only recently diverged from a common ancestor (Hambäck et al. 2013; Fors et al. 2014). The parasitism rate on the two host species varies between localities but in general, *G. pusilla* experiences a lower parasitism rate than *G. calmariensis* if they co-occur (Fors et al. 2014). Both encapsulation and melanization events are very common in *G. pusilla* larvae infected by wasps, whereas in *G. calmariensis*, capsule formation and melanization of wasp eggs are rarely observed. If encapsulation takes place successfully upon parasitism, the capsule formation usually starts within 4–6 h and is completed after ∼48 h (Fors et al. 2014). During encapsulation process, the capsule is often melanized and melanized parasitoid eggs can be clearly observed upon digestion after 48 h. Wound healing usually occurs quickly on the wound sites within the first few hours upon parasitism, where melanization also plays a role in the blackening of wound. In general, *G. pusilla* exerts a more potent immune response against *A. parviclava* than *G. calmariensis*, with cellular level investigations documenting that two classes of hemocytes involved in encapsulation, phagocytes and lamellocytes, increased their production upon infection in *G. pusilla* (Fors et al. 2014). In order to move beyond cellular phenotype level study, here we examined differences in the gene expression between these two species, using a time-course sampling design following parasitoid attack. Beyond whole-transcriptome level analyses, we also focused upon annotated immune genes based on previous knowledge from *D. melanogaster* and other arthropod species (Wertheim et al. 2005; Tribolium Genome Sequencing Consortium 2008). Based on the previous work, we expected more immune genes, especially hematopoiesis genes, to be differentially expressed following parasitoid attack in the species with the strong immune response, *G. pusilla*.

## Materials and Methods

### Study Species

*Galerucella pusilla* and *G. calmariensis* have similar life cycles. They overwinter as adults and appear in May in the study area, and soon start to lay eggs on the leaves or stem of the *L. salicaria* host plants. It takes a few weeks for the eggs to hatch, 2–3 weeks for the larvae to pupate, and another 2–3 weeks for the adults to emerge from the pupae. In Sweden, *G. pusilla* occurs only in the south and up to central Sweden until Sundsvall (N62°, E17°), whereas *G. calmariensis* is also distributed further north (Fors et al. 2014). Despite the morphological similarity between the species, they can be distinguished based on several traits. The larvae of *G. calmariensis* usually have a bright yellow color, whereas the larvae of *G. pusilla* tend to have a lighter yellow tone (Hambäck 2004), and adult males can be distinguished by the presence of spurs on the meso- and metatibiae (Cossé 2004).

*Asecodes parviclava* is a small (<1 mm) endoparasitoid wasp that lays eggs in the larvae of *G. pusilla* and *G. calmariensis*, as well as in an additional species, *G. tenella*. Once successfully parasitized, parasitoid larvae consume the interior of the host larvae and hinder pupation. During the following summer, the adult parasitoids hatch and subsequently emerge from the host mummy (Hansson and Hambäck 2013). The parasitism rate on the two host species varies between localities but in general, *G. pusilla* experiences a lower parasitism rate than *G. calmariensis* if they co-occur (Fors et al. 2014). Both encapsulation and melanization events are very common in *G. pusilla* larvae infected by wasps, whereas in *G. calmariensis*, capsule formation and melanization of wasp eggs are rarely observed. If encapsulation takes place successfully upon parasitism, the capsule formation usually starts within 4–6 h and is completed after ∼48 h (Fors et al. 2014). During encapsulation process, the capsule is often melanized and melanized parasitoid eggs can be clearly observed upon digestion after 48 h. Wound healing usually occurs quickly on the wound sites within the first few hours upon parasitism, where melanization also plays a role in the blackening of wound.

For our experiments, adult beetles were collected in the field at Iggön (60°52′18″N, 17°19′29″E), where both species co-occur in mid-May, and were reared in the laboratory at room temperature. For each beetle species, six adults of both sexes were placed on a *L. salicaria* plant in a transparent plastic cage. Beetles mated randomly and laid eggs on the plant leaves. Newly hatched larvae of approximately the same size were removed from the cages for use in the parasitism experiment when they had reached the second instar. The female parasitoids for parasitism experiments were sampled from previous season’s parasitized pupae. Parasitoids were collected from *G. calmariensis* in a different area with very high parasitism rates: Ällön (63°13′46″N, 19°4′59″E).

### Parasitism Experiment

To investigate the time course of the immune response of the two species, we performed an infection experiment in 2013 and 2014. All samples were prepared in 2014 except for the 12-h posttreatment groups (control and infection), which were performed in 2013. For the infection treatment, eight laboratory-reared second-instar beetle larvae of the same species were placed in a 200-ml transparent plastic container together with four *A. parviclava* females for 4 h to ensure sufficient time for parasitism. We visually confirmed that all the wasps mounted multiple attacks within the first 10 min and became much less active after 1 h. Parasitized larvae were subsequently transferred to new petri dishes for 1, 4, and 12 h to allow host immune responses. The control groups were treated in the same way, except that no parasitoids were introduced. Three biological replicates, each consisting of eight individual larvae, were generated in each treatment group in *G. pusilla*, whereas the number of biological replicates varied between two to four in *G. calmariensis* (supplementary fig. S1, Supplementary Material online). In total, across all treatments, 18 samples for each *Galerucella* species were prepared for extraction and sequencing.

### Extraction and Sequencing

The eight larvae for each replicate were pooled in one Eppendorf tube, snap frozen in liquid nitrogen, and stored at −80 °. All samples were homogenized and total RNA was extracted using TRIzol LS Reagent (Invitrogen) following the manufacturer’s instructions. A quality check was performed with a NanoDrop spectrophotometer and a 2100 Bioanalyzer. Only samples with a 260/280 and 260/230 close to or above two and with no severe degradation visible on the Aligent RNA 600 Nano Assay electropherogram were processed to library preparation. All 36 samples (as shown in supplementary fig. S1, Supplementary Material online) were sent to SciLifeLab Stockholm for library preparation and sequencing. Specifically, 26 libraries from 1- and 4-h posttreatment groups in both species were prepared once and sequenced in two lanes on the same flowcell, whereas ten libraries from 12-h treatment groups were sequenced in one lane. Poly-A-enriched RNA libraries were constructed and only libraries with a concentration >10 nM were paired-end 2× 125-bp sequenced on a HiSeq 2500 (Illumina) platform.

Variation in gene expression between replicates may have occurred for reasons that we were unable to control. For instance, we were unable to control the number of eggs laid by female parasitoids in the experiment, which may have led to variation in both the strength and timing of infection at the individual level and thus to variations in the immune gene expression. Moreover, the expression levels of significant immune-related genes could have been diluted by pooling eight individuals in each sample, or by our use of the whole larvae rather than specific gland tissues for RNA sampling.

### Transcriptome Assembly and Filtering

In total, 337 million *G. pusilla* and 310 million *G. calmariensis* pair-ended raw reads from all libraries were first processed to Stacks 2.0b through clonefilter to remove polymerase chain reaction duplicates (Catchen et al. 2013). Ribosomal RNA were removed with SortMeRNA v2.1 (Kopylova et al. 2012), after which Trimmomatic v0.32 (Bolger et al. 2014) were performed to remove adapters and low quality reads, leading to 271 and 224 million paired-ended reads for *G. pusilla* and *G. calmariensis*, respectively.

We performed quality control using FastQC v0.11.5 (Babraham Bioinformatics 2011) before and after filtering. Only reads ≥40 bp and with a quality score higher than 25 in both read start and end were retained. Clean reads of both species were normalized using TRINITY v2.1.0 in silico *normalization* (Grabherr et al. 2011) to remove large excess of reads corresponding to moderately and highly expressed transcripts. The resulting data were merged and normalized again prior to assembly. De novo transcriptome assembly of reads was done using TRINITY 2.1.0 (Grabherr et al. 2011) with the default parameters. In total, 195,422 putative genes and 262,505 transcripts were generated for *G. pusilla*, whereas 224,904 putative genes and 300,593 transcripts were obtained for *G. calmariensis*.

To further filter the transcriptomes, we ran DETONATE v1.11 (Li et al. 2014) to calculate model-based scores that describe how well the contigs were supported by the RNA-Seq data. In total, 42,166 and 57,165 contigs with negative scores across all samples were trimmed from the *G. pusilla* and *G. calmariensis* raw assembly, respectively.

In order to remove the redundancy resulting from the transcriptome assembly and to improve the quality of the transcripts, the transcriptome assembly was processed with the EvidentialGene tr2aacds pipeline (Nakasugi et al. 2014). The pipeline started with inferring coding DNA sequence (CDS) and amino acid sequence for each contig, and then fastanrdb, CD-HIT-EST, and BLAST were run to select the best CDS based on the identity and alignment to each other. The output set of transcripts were classified as “main,” “alternate,” and “dropped,” which did not pass the filters (Nakasugi et al. 2014; Postnikova et al. 2015). Only the “main” set of de novo assemblies were used in subsequent analyses. To filter contamination from the assembly, we ran Kraken v1.0 (Wood and Salzberg 2014) to remove potential bacteria, archaea, virus, human, and plant sources (downloaded from RefSeq September 2017). In total, 484 and 528 contaminant contigs from the *G. pusilla* and *G. calmariensis* were removed.

Finally, we filtered potential parasitoid genes by mapping the transcriptomes to *Nasonia vitripennis* proteome and removing reads that uniquely mapped to *Nasonia vitripennis* (coverage 99.5% as the cutoff). This procedure identified 164 and 165 candidate parasitoid genes from *G. pusilla* and *G. calmariensis* assembly, respectively. Finally, we only retained genes that are expressed in infection-treated samples because wasp genes are not supposed to exist in the uninfected samples, which led to 144 and 145 parasitoid genes. No differential expression pattern was observed in these genes between any pairwise comparisons in the treatment groups.

### Quality Assessment and Annotation of Transcriptome Assembly

We used Benchmarking Universal Single-Copy Orthologs (BUSCO) from OrthoDB (_v1.22) (Simão et al. 2015) to measure the completeness of de novo assembled transcriptomes by searching for lineage-specific conserved single-copy orthologs. BUSCO assembly assessment first identifies candidate regions from the genome/transcriptome to be assessed with TBlastN searches using BUSCO consensus sequences. Gene structures are then predicted and assessed using HMMER and lineage-specific BUSCO profiles (in our case, arthropods BUSCO profiles as the database) to classify matches as complete, duplicated, fragmented, or missing. This tool provides a genome-free/reference-free validation of transcriptome assembly completeness and allows comparison between multiple assemblies.

We aligned our CDS against the existing *D. melanogaster* proteome (downloaded from NCBI in 2017) and vice versa using BlastX v2.5.0+ with an *e*-value ≤10^−5^ cutoff to compare the presence of genes in our assembly with *D. melanogaster*. We also compared our CDS with the closer relative *Tribolium castaneum* using the same approach and identified candidate immune genes in general (supplementary table S3, Supplementary Material online) based on orthologs in *Tribolium* (Zou et al. 2007). To gain a better understanding of the similarity and differences of the species, we performed reciprocal blast between the two *Galerucella* species in both whole-transcriptome level and highly expressed transcripts which have average read counts higher than 10 across all the samples.

In order to expand the functional inferences to a broader scale, EggNOG v4.5 (Huerta-cepas et al. 2016) was used to assign functional annotation and Gene Ontology (GO) terms to the transcripts. Transcripts were first translated to amino acids and then sent to EggNOG with *Arthropoda* as taxonomic scope and DIAMOND as search program.

### Differential Expression Analysis

All samples were mapped to the reference transcriptomes by Salmon v0.9.1 with specific flags of “–gcbias and –seqbias” (Patro et al. 2017). Mapping rates were similar between samples (77% to 82%). A matrix containing weighted read counts assigned to gene *i* in sample *j* was generated and transformed using “Relative Log Expression” normalization implemented in Deseq2 (Love et al. 2014). Deseq2 was then used to test for differential gene expression through the use of negative binomial generalized linear models. Libraries derived from the same samples (*N* = 12 for *G. pusilla* and *N* = 14 for *G. calmariensis*, two technical replicates for each sample) were first collapsed through “-collapseReplicates” function. Because samples from 12 h were collected and sequenced in a different year, we analyzed 12-h samples separately from others (1 and 4 h), to ensure that the detected expression variation is truly biologically relevant and not due to differences in experimental procedures, handling or sequencing between sampling years. Specifically, in the data pool of 1- and 4-h samples, treatments were compared with each other correcting for variation arising from Time, and Treatment-by-Time. We used a likelihood ratio test to determine if the Treatment alone induces a change in gene expression at any point in time by comparing the full model (∼Time+ Treatment + Time:Treatment) against the reduced model (∼Time + Treatment). For 12-h samples, we applied the full model (∼Treatment) against the reduced model (∼1) because only one time point is included in the data set. *P* values were adjusted for multiple testing to control false discovery rate (FDR) (Benjamini and Hochberg 1995) and only transcripts with an adjusted *P* value <0.05 were classified as differentially expressed.

GO enrichment test of biological process categories was performed by BIOCONDUCTOR package topGO (Alexa and Rahnenfuhrer 2010). The background genes included in the GO enrichment test were those transcripts with existing GO terms in our EggNOG annotation. Differentially expressed genes (DEGs) from each time-course comparisons when then tested for enrichment against the background based on Fisher’s exact test (*P* < 0.05). Afterward, enriched GO term lists were simplified and clustered to representative functional subsets using REVIGO *Drosophila* database (Supek et al. 2011). Finally, we searched in our annotations for a subset of genes known to be important in immune response against parasitoid wasp attack in *Drosophila*. This includes a list of 166 genes involved in signaling, effector, protease, recognition, wound healing, melanization, hemocyte (hemocyte proliferation and differentiation), and hemocyte regulation (regulation of differentiation and proliferation) (Zettervall et al. 2004; Salazar-Jaramillo et al. 2014). In order to explore the presence and variation of immune-related genes in both transcriptome assemblies, we searched the immune genes in our *Drosophila*-based annotation. We also examined our data set to determine if any of these genes were differentially expressed.

## Results

### Transcriptome Sequencing and Assembly

In total, we sequenced 18 pooled RNA-Seq libraries for both *G. pusilla* and *G. calmariensis*. After polymerase chain reaction duplication removal, ribosomal RNA filtering, adapter removal, and quality trimming, we obtained 271 million and 224 million paired-ended reads for *G. pusilla* and *G. calmariensis*, respectively. As there is no sequenced *Galerucella* genome, we first assembled transcriptomes de novo for our two study species using TRINITY. De novo transcriptome assemblies were based on all the data for each species and yielded 262,505 transcript isoforms representing 195,422 unique transcripts (N50 = 1,428) for *G. pusilla* and 300,593 isoforms representing 224,904 unique transcripts for *G. calmariensis* (N50 = 1,070), before filtering. After removing transcripts with poor read support, filtering contaminant sequences, and selecting representative transcript isoforms, we retained 52,563 transcripts for *G. pusilla* and 59,781 transcripts for *G. calmariensis* (table 1). Reciprocal blast identified 48,333 transcripts that are overlapping between *G. pusilla* (84.4%) and *G. calmariensis* (75.5%). After filtering out, lowly expressed transcripts with average read counts lower than 10, 16,914 transcripts from *G. pusilla* and 16,713 transcripts from *G. calmariensis* were retained, respectively, among which 15,996 transcripts were overlapping between the two species.

**Table 1:** Summary of *Galerucella pusilla* and *Galerucella calmariensis* Reference Transcriptomes

Species	*G. pusilla*	*G. calmariensis*
Summary of transcriptomes		
Total transcripts	52,563	59,781
Total isoforms	57,255	64,000
N50 (bp)	1,840	1,574
Average contig length (bp)	1,001	899
BUSCO results		
Complete BUSCO hits (%)	1,006/94.4%	1,011/94.8%
Complete single hits	774/72.6%	751/70.5%
Duplicated	232	260
Fragment	36	35
Missing	24	20
Reciprocal BLAST hits with *Drosophila* (13,469 genes)	10,255/76.1%	10,708/79.5%
Reciprocal BLAST hits with *Tribolium* (16,563 genes)	11,402/68.8%	11,425/69.0%
EggNOG hits with *Arthropoda*	21,487	22,939
GO terms with *Arthropoda*	6,963	7,422

Note.—The assembled references were compared with *Drosophila melanogaster*, *Tribolium castaneum*, and BUSCO arthropod database for quality assessment.

Comparing our de novo assembled transcriptomes to the *D. melanogaster* proteome using BlastX gave significant hits (*e*-value <10^−5^) for 16,125 transcripts (28.2%) from *G. pusilla* and 17,229 (26.9%) transcripts from *G. calmariensis*. To verify the blast results, we performed reciprocal BLAST of our assembly against the model organisms *D. melanogaster* and *T. castaneum*. Slightly more BLAST hits were identified in *G. calmariensis* than in *G. pusilla* when comparing with both *Drosophila* and *Tribolium*. To expand the functional inferences to a broader scale, we used EggNOG mapper to identify and assign functional GO terms to the transcripts using an *Arthropoda* database (supplementary fig. S2, Supplementary Material online). We measured the completeness of these transcripts using the BUSCO arthropod database and found complete matches to more than 94% of BUSCO genes in both (table 1), indicating that our de novo transcriptomes were relatively complete with respect to core arthropod gene content.

### Differential Expression

To test whether the transcriptional response to parasitoid infection differed between *G. calmariensis* and *G. pusilla*, we examined changes in gene expression between species and time points. We used Deseq2 with an FDR adjusted *P* < 0.05 cutoff and compared the log 2 fold change over average expression strength between infected and noninfected conditions. We tabulated the numbers of significant DEGs for four treatment comparisons between different time points in both beetle species: 1-h postinfection versus 1-h control, 4-h postinfection versus 4-h control, treatment-specific DEGs from 1 to 4 h, and 12-h postinfection versus 12-h control (table 2).

**Table 2:** Number of DEGs in *Galerucella pusilla* and *Galerucella calmariensis* after Wasp Attack (FDR < 0.05) at Different Time Points

	*G. pusilla*				*G. calmariensis*			
	1-h Treat versus 1-h Control	4-h Treat versus 4-h Control	4-h Treat versus 1-h Treat	12-h Treat versus 12-h Control	1-h Treat versus 1-h Control	4-h Treat versus 4-h Control	4-h Treat versus 1-h Treat	12-h Treat versus 12-h Control
No. of upregulated genes	58	985	828	775	169	984	394	46
No. of downregulated genes	32	528	416	1,465	8	126	38	6
Total no. of DEGs	90	1,513	1,244	2,240	177	1,110	432	52

Generally, more genes were differentially expressed in response to parasitoid infection in *G. pusilla* than in *G. calmariensis* for all time points except 1-h postinfection. This discrepancy increased at later time points and the largest difference was found 12 h after parasitoid attack (table 2), mainly because the number of DEGs increased over time in *G. pusilla* but decreased strongly between 4 and 12 h for *G. calmariensis*. This pattern was also driven by the number of downregulated DEGs that decreased faster in *G. calmariensis*, already at 4 h, compared with *G. pusilla*.

### Functional Inferences of Overrepresented DEGs

To place the expression differences between the two species and different time points in a biologically meaningful context, we performed GO enrichment tests to determine whether these DEGs are overly represented in certain biological process categories. We identified top overrepresented biological function categories among both up- and down-regulated DEGs in the two species at different time points (supplementary table S1 and fig. S2, Supplementary Material online).

In summary, at the molecular level, there was an enrichment of both up- and down-regulated DEGs with functions related to energy production, biosynthetic process, and metabolic process in both species. The main difference between the resistant species *G. pusilla* and the susceptible species *G. calmariensis* appears to be the sustained and substantial immune response and wound healing process mounted by *G. pusilla* larvae.

In *G. pusilla*, the most common response to wasp attack throughout all the time points involved immune system process/innate immune response (supplementary table S1 and figs. S2 and S3, Supplementary Material online). Another biological processes that started at an early stage (1 h), which corresponds to upregulated DEGs upon parasitoid infection, were energy related (ATPase activity, mitochondrial ATP synthesis coupled proton transport). This suggests that immune response upon infection in *G. pusilla* may be associated with a metabolic switch that enables rapid production of ATP. Two biological function categories, cuticle development and chitin metabolic, which are considered to play important roles in wound healing responses, were highly enriched due to the upregulated DEGs in that time point when comparing 1-h versus 4-h postinfection and when comparing 4-h postinfection versus 4-h control in *G. pusilla*. The large set of downregulated genes at 4 h have broad and vague function (e.g., RNA processing, water transport, renal system process, and mRNA metabolic process), and it is hard to link this subset of genes to any informative biological events. At 12-h postinfection, regulation of immune response became the most enriched process, with contributions mainly from upregulated genes and partially from downregulated genes.

In *G. calmariensis*, general biological functional groups such as biosynthetic process and metabolic process were significantly overrepresented at all the time points.

GO terms related to energy generation (mitochondrial ATP synthesis coupled proton transport, regulation of hippo signaling) were also enriched at 4-h and 12-h time points.

However, immune-related functional categories were rarely overrepresented in *G. calmariensis* except the enrichment of negative regulation of immune system processes at 12-h postparasitism (supplementary table S1, Supplementary Material online).

### Candidate Antiparasitoid Immune-Related Genes

**Fig. 1.**
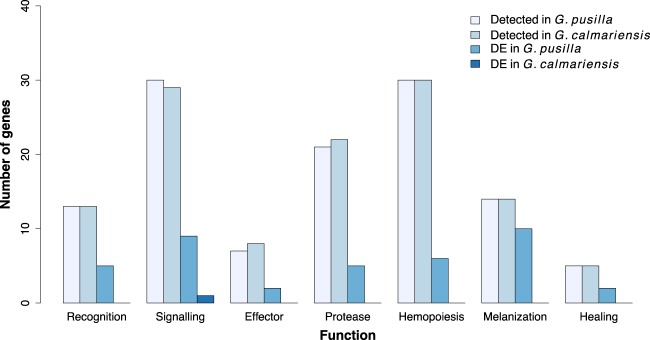
—Numbers of candidate immune genes detected and differentially expressed in *G. pusilla* and *G. calmariensis* in at least one time point postinfection, based on immune function identified from *D. melanogaster*. There is an enrichment of immune genes among all the DEGs in all the time points after parasitism in *G. pusilla* (Fisher’s exact test, *P* < 0.05).

To test whether immune-related genes, and particularly hematopoiesis genes, in *G. pusilla* showed a stronger transcriptional response to parasitoid infection than *G. calmariensis*, we first identified coleopteran candidate immune genes from blast matches with the complete set of *Drosophila* immune genes (Wertheim et al. 2005; Schlenke et al. 2007; Salazar-Jaramillo et al. 2014). This set was composed of 166 protein-coding genes, which were classified according to the seven categories for immune-related genes detected in *Drosophila*: recognition, signaling, effector, proteases, hematopoiesis, melanization, and wound healing (supplementary table S2, Supplementary Material online). More than half of the effector genes in *D. melanogaster* were not detected in either of the two *Galerucella* species whereas most signaling, hematopoiesis, and melanization genes in *Drosophila* were detectably expressed in both *Galerucella* species. Specifically, among the 166 candidate genes, 120 and 121 genes were detected in the *G. pusilla* and *G. calmariensis* transcriptomes, respectively (fig. 1 and supplementary table S2, Supplementary Material online). Most of these genes were shared between *G. pusilla* and *G. calmariensis*, and only five genes involved in signaling, effector, and protease were expressed only in one species. None of these five genes were differentially expressed at any time point and are not discussed further (supplementary table S2, Supplementary Material online).

When comparing the differential expression of immune-related genes in response to parasitoid attack between species, we found great differences in the number of DEGs between *G. pusilla* and *G. calmariensis*. Among the 120 immune-related genes detected in *G. pusilla* transcriptome, 39 (32.5%) genes were differentially expressed between the infected and the noninfected group in at least one time point. These were similarly distributed between all immune-related functions (fig. 1). In contrast, only one gene (0.8%) in the *G. calmariensis* transcriptome was differentially expressed between infected and noninfected groups. The expression profiles of all differentially expressed immune genes are provided in supplementary table S2, Supplementary Material online. We also performed a candidate immune gene search by comparing with *Tribolium* in order to obtain a more complete data set because they are more closely related to our organism. A list of 217 immune genes in general in *Tribolium* (Zou et al. 2007) was mapped with our beetle transcriptome, and 135 genes from *G. pusilla* and 128 genes from *G. calmariensis* were identified. Similar expression pattern was observed as for immune genes characterized from *Drosophila* that immune genes responded more strongly in *G. pusilla* than in *G. calmariensis* (supplementary table S3, Supplementary Material online) and further discussion will only be based on *Drosophila* data sets.

**Fig. 2.**
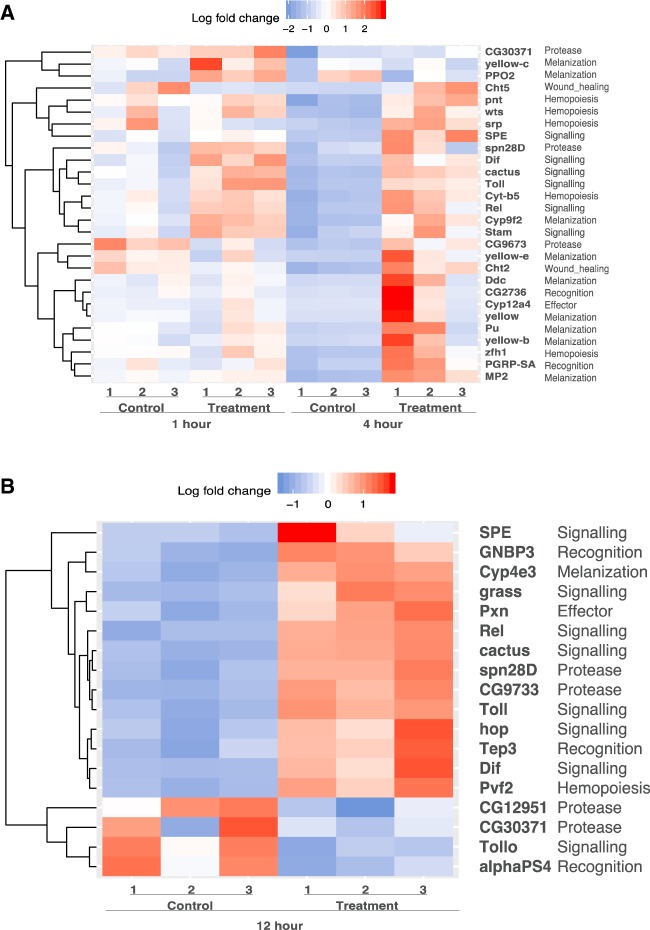
—Heat maps displaying immune-related genes differentially expressed in *G. pusilla* across different time points (*A*: 1 and 4 h, *n *=* *28; *B*: 12 h, *n *=* *18) between samples infected by parasitoid wasp *A. parviclava* and control groups. For each DEG, we present the log fold change in each treatment sample, the name of the gene, and the immune function classification. Red blocks represent genes that are upregulated in the corresponding treatment, whereas blue blocks correspond to downregulation.

Twenty-four immune-related genes were differentially expressed 4 h after parasitoid infection in *G. pusilla*, whereas only one was detected in *G. calmariensis* (fig. 2 and supplementary fig. S4 and table S2, Supplementary Material online). Immune-related DEGs in *G. pusilla* included important genes involved in parasitoid recognition (*CG2736* and *PGRP-SA*), Toll, IMD, and JAK-STAT signaling pathways (*Rel*, *SPE*, *Toll*, and *cactus*), effector with oxidoreductase activity (*Cyp12a4*), hemocyte development and proliferation (*wts*), plasmatocyte differentiation (*pnt*), and lamellocyte differentiation (*srp*, *zfh1*, and *Cyt-b5*). Several serine-type protease, prophenoloxidase, and members of yellow gene family (*Cyp9f2*, *Ddc*, *MP2*, *PPO2*, *Pu*, *yellow*, *yellow-e*, and *yellow-b*), which exhibit activation in the melanization cascade, were differentially expressed at 4 h. However, some key genes involved in melanin deposition such as the immunoglobulin-like protein *PPO3* and a serine-type endopeptidase (*CG11313*) showed no induction in our profile. Two wound healing genes with chitinase activity (*Cht2*, *Cht5*) showed strong signals of differential expression at the 4-h time point in *G. pusilla*, which is in accordance with GO biological process inferences. We also tested for time-course expression differences between 1- and 4-h time interval and detected less DEGs compared with the expression patterns at 4-h time point (4-h postinfection versus 4-h control), indicating an immediate activation of immune genes in *G. pusilla*. Thirteen genes were differentially expressed from 1 to 4 h, among which 10 (76.9%) genes overlap with the list of DEGs between 4-h treatment and 4-h control.

In contrast to *G. pusilla*, only one immune gene was differentially expressed in *G. calmariensis* for any time point. This was a signal transducing adaptor molecule (stam) which encodes a component of the ESCRT-0 complex and is considered to play a role in JAK-STAT signaling.

**Fig. 3.**
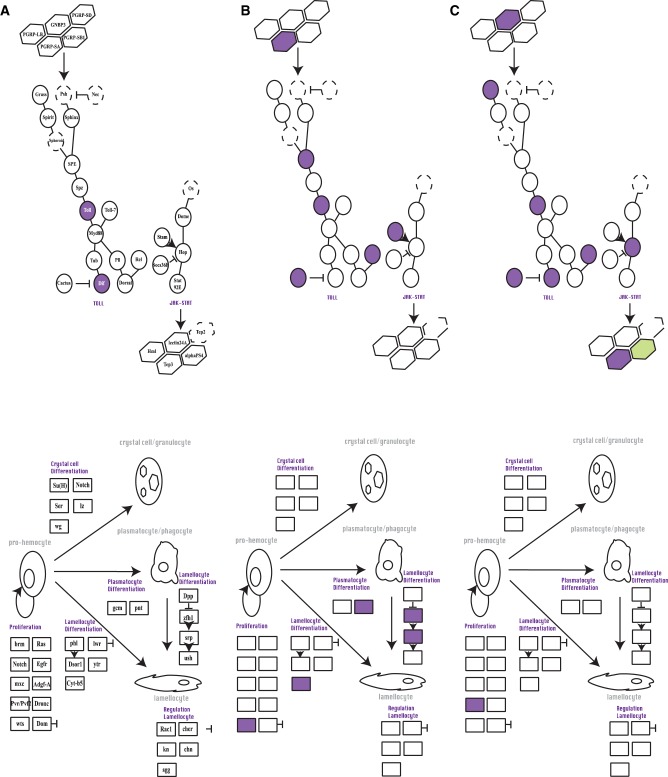
—Schematic representation of immune pathways expressed under parasitoid attack in *G. pusilla* at different time points after parasitoid attack: (*A*) 1 h, (*B*) 4 h, and (*C*) 12 h. Dashed-line circles correspond to proteins known to be in the pathway in *Drosophila* but which were not detected as being expressed in *Galerucella*. Upper half illustrates pathways associated with recognition and signaling pathways, whereas the lower half represents genes involved in hemopoiesis. Purple circles represent genes that were upregulated in the infected samples compared with noninfected ones, whereas green colors correspond to downregulated DEGs. Gene names are presented in (*A*).

We observed temporal gene expression responses to parasitoid infection in *G. pusilla* by comparing expression patterns at three time points (figs. 2 and 3). Most signaling genes, especially genes involved in Toll signaling pathway, were differentially expressed already at an early stage (4 h) after parasitism and remained active until 12 h. Fewer genes were induced by parasitoid infection at 12 h compared with 4 h. Genes involved in hematopoiesis, melanization, and wound healing were only activated after 4-h postparasitism, followed by decrease of signals at 12 h.

Less variation of DEGs was observed in *G. calmariensis* between time points (supplementary table S2, Supplementary Material online). In general, immune-related genes showed much less differential expression in all gene categories for *G. calmariensis* than for *G. pusilla*. There was a drastic contrast in the genes involved in encapsulation processes between 4- and 12-h postparasitism (supplementary table S2, Supplementary Material online).

## Discussion

We assembled de novo transcriptomes of two closely related chrysomelid beetles with contrasting immunocompetence: *G. pusilla* and *G. calmariensis*, which provide new genetic resources for future analysis of Coleoptera species. Previous studies investigated the cellular immune competence between these two species, identifying a better performance of *G. pusilla* than *G. calmariensis* in encapsulation and hematopoiesis processes (Hambäck et al. 2013; Fors et al. 2014). To understand the role of gene regulatory differences for such contrasting immunocompetence, we compared gene expression patterns of the two *Galerucella* species at three time points after infection by *A. parviclava*, identifying important biological processes that are overrepresented upon parasitism. We then focused on immune candidate genes, finding similar numbers of immune-related genes in both beetle transcriptomes, with significantly more of these genes induced in *G. pusilla* than in *G. calmariensis*, especially in genes involved in immune signaling, hematopoiesis, and melanization.

Our data show that *G. pusilla* mount a strong host immune transcriptional reaction incorporating both humoral and cellular responses (supplementary table S1, Supplementary Material online). Functional categories related to energy allocation (the regulation of ATPase activity and mitochondrial ATP synthesis) also appear to be enriched at an early stage upon parasitism, which play a role in energy generation to help the larvae to pay the cost of follow-up immune responses. In contrast to *G. pusilla*, *G. calmariensis* appears to completely fail to defend themselves against wasp attack, as if these parasitoid intruders were never recognized. Another possibility could be the suppression of the immune system by the parasitoid, as they inject virulent substances such as ovarian protein venoms or virus-like particles into the host along with the wasp eggs at the time of oviposition. Consistent with this suggestion, we find an enrichment of GO term “negative regulation of immune system” in upregulated DEGs at 12-h postparasitism in *G. calmariensis*.

The time point where most hematopoiesis genes showed the strongest induction in the larvae was 4 h after parasitism. This observation is in line with previous findings in *Drosophila* where the proliferation and differentiation of hemocytes peaked at the same time point (Wertheim et al. 2005). The genes that were differentially expressed at 4 h in *G. pusilla* include a broad range of functions, such as hemocyte proliferation, lamellocyte differentiation, and plasmatocyte differentiation. In particular, we found an upregulation of *Cyt-b5* which encodes a conserved hemoprotein that promotes the differentiation of precursor hemocytes (Frandsen et al. 2008). Other DEGs found in the *G. pusilla* expression profile are responsible for lamellocyte differentiation. For instance, *zfh1* and *srp* are elements in a transcription factor cascade that play a role as the switch between plasmatocyte and lamellocyte fate (Frandsen et al. 2008). In addition, some genes such as *wts* in the hippo pathway are known as key regulators of the differentiation of lamellocytes and crystal cells in *Drosophila* (Milton et al. 2014), where their upregulation results in the differentiation of lamellocytes and downregulation promotes crystal cell differentiation. The upregulation of the *pnt* gene in our expression profile is expected to regulate prohemocyte differentiation and the production of plasmatocytes. Several genes involved in melanization (i.e., Serine proteases, Prophenoloxidases, and yellow gene family) were only highly activated at 4-h postparasitism. As is reported in a conservative genome-wide gene expression profiling in response to parasitoid attack in by Wertheim et al. (2005), capsule melanization in *Drosophila* usually takes place 24–72 h after parasitism. This late response suggests that the massive overexpression of melanization genes after 4 h in *G. pusilla* may only be a healing response to the wound sites caused by the wasp ovipositor, and not an immune response against parasitoid eggs. This interpretation is further supported by the fact that the biological functions of cuticle development and chitin metabolism are highly enriched at 4-h postinfection in *G. pusilla*.

Although our results generally matched the differential expression profiles observed in previous studies on immune response of *Drosophila* larvae upon parasitoid attack, we also observed differences. For instance, the *cactus* gene which acts as a negative regulator of the Toll signaling pathway (Evans et al. 2003) was consistently upregulated in our time-course profile in *G. pusilla*. A probable explanation for this pattern may be differences in the use of immune pathways in *G. pusilla* compared with *Drosophila*, away from Toll signaling and toward hemocyte proliferation. We also found downregulation of some genes that have been implicated in melanization (*PPO2*, *yellow-c*), which may be explained by experimental variation (in particular for early phases of the response, see Material and Methods) or by species-specific differences in immune regulation and allocation.

Although we identified *Galerucella* homologs for a wide array of *Drosophila* immune genes and provide molecular support for the previously reported difference in immune competence of *G. calmariensis* and *G. pusilla*, there are limitations. First, we were unable to annotate fast evolving genes, among which will be many immune genes such as antimicrobial peptides (Sackton et al. 2007). Also, previous work in *Drosophila* has suggested that effector and protease proteins may rapidly diversify as a result of duplication events during host–parasite coevolutionary process (Sackton et al. 2007; Waterhouse et al. 2007; Salazar-Jaramillo et al. 2014), even between closely related species, and if this duplication is common also in our taxa, our ability to correctly annotate these genes could be limited. Second, identifying real orthologs of multigene families is difficult and even when possible may not reveal their true function. This may be the case for some of effectors, members of the hexamerin family, including phenoloxidases and for regulators of cellular immunity. Third, immune effectors would have been undetected if they were neither induced upon wasp infestation nor expressed constitutively.

## Conclusion

Through a combination of de novo assembly and differential expression analysis, we elucidate regulatory differences underlying the contrasting immunocompetence between two closely related *Galerucella* species. We characterize specific genes and pathways that play roles in different immune functions induced by parasitoid attack and suggest that a rechanneling of the hematopoiesis process is the main reason for inefficient host immunity in *Galerucella* against parasitoid wasps. These findings now provide a list of specific genes and functions that can be further investigated for their role in responding parasitoid attack.

### Ethics Approval and Consent to Participate

No endangered or protected species were involved in this study. All sites used for field collection are located on privately owned land. According to Swedish law, no permission is needed from private land owners for field sampling. 

### Consent for Publication

Not applicable.

## Supplementary Material

evaa075_Supplementary_Data

## Notes

**Data deposition:** Sequence data generated and analyzed during the current study have been deposited at the European Nucleotide Archive (http://www.ebi.ac.uk/ena) under the accession PRJEB28202.
