# Supplementary material for: Differential Expression of Immune Genes between Two Closely Related Beetle Species with Different Immunocompetence following Attack by Asecodes parviclava
Source: Genome Biol Evol. 2020 Apr 13;12(5):522–34. doi: 10.1093/gbe/evaa075 (PMC7211424; doi:10.1093/gbe/evaa075)

Figure S1. Summary of experimental design for samples sequenced in RNAseq.
All samples were prepared in 2014 except for the 12 hours post-treatment groups (control and infection), which were performed in 2013. Two technical replicates, which come from the same library that were aliquoted and sequenced into two lanes on the same flowcell, were generated for 1 hour and 4 hour samples.

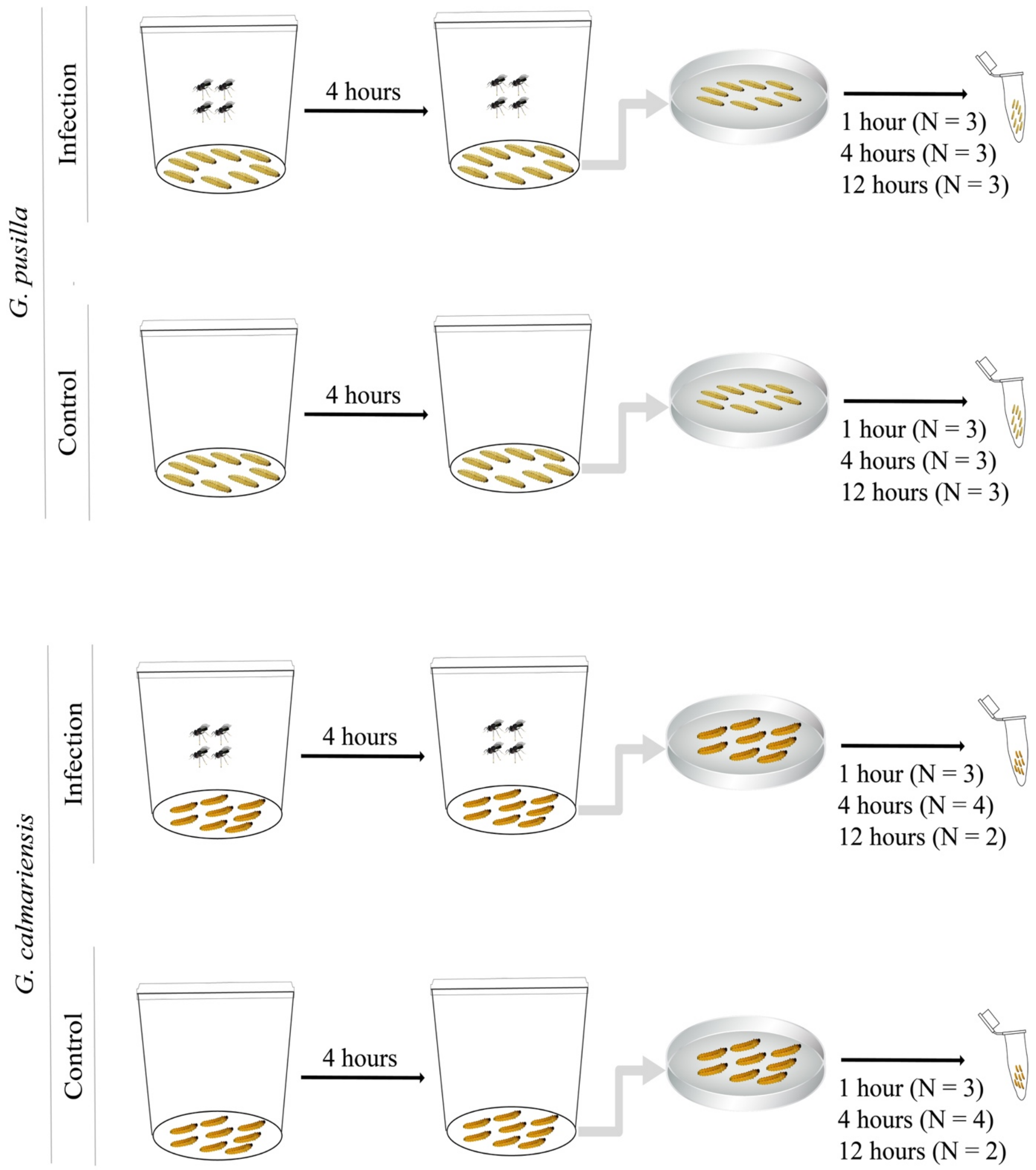

Supplement: evaa075_Supplementary_Data [file evaa075_supplementary_data.zip › Figure S1.pdf]
