# Supplementary material for: Differential Expression of Immune Genes between Two Closely Related Beetle Species with Different Immunocompetence following Attack by Asecodes parviclava
Source: Genome Biol Evol. 2020 Apr 13;12(5):522–34. doi: 10.1093/gbe/evaa075 (PMC7211424; doi:10.1093/gbe/evaa075)

Figure S2. REVIGO treemap visualization of over-represented GO terms with biological processes at different time points post-infection in two *Gallerucella* species.

TopGO was used to identify GO Biological Processes that were over-represented among DEGs at an FDR cutoff of 0.05. Enriched GO terms with p values of less than 0.05 were used to generate a treemap with biological processes. The size of each color block is proportional to the p value for that category.

1.1 GO terms inferred from all DEGs 1 hour post-infection in *G. calmariensis*

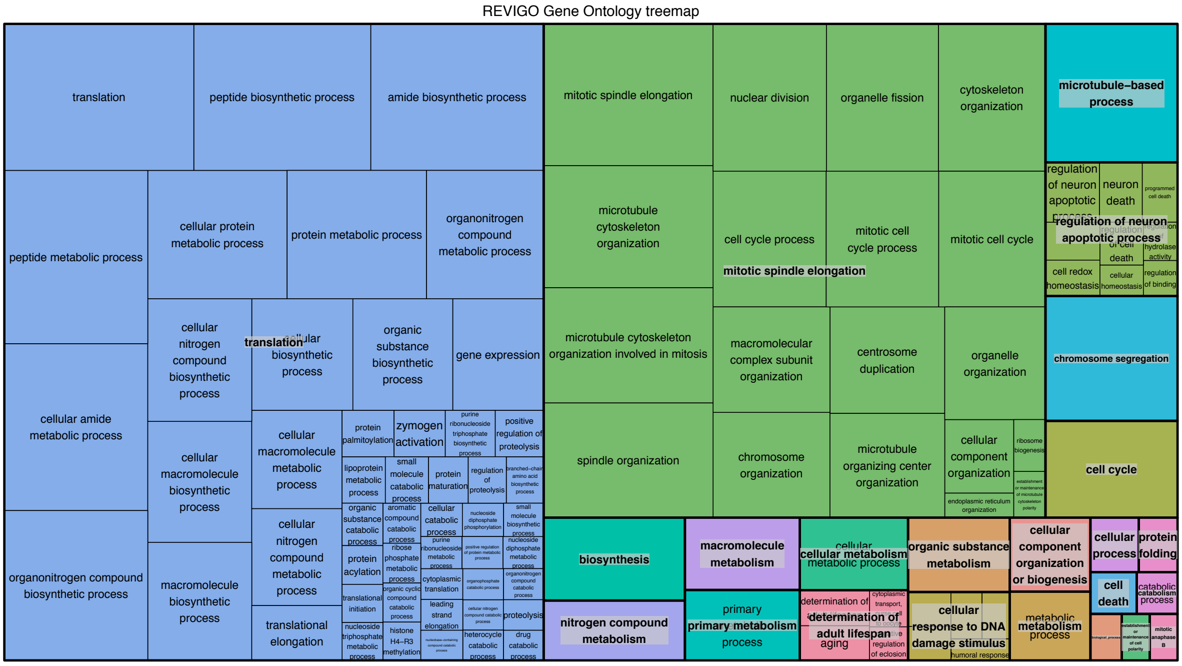

1.2 GO terms inferred from up-regulated DEGs 1 hour post-infection in *G. calmariensis*

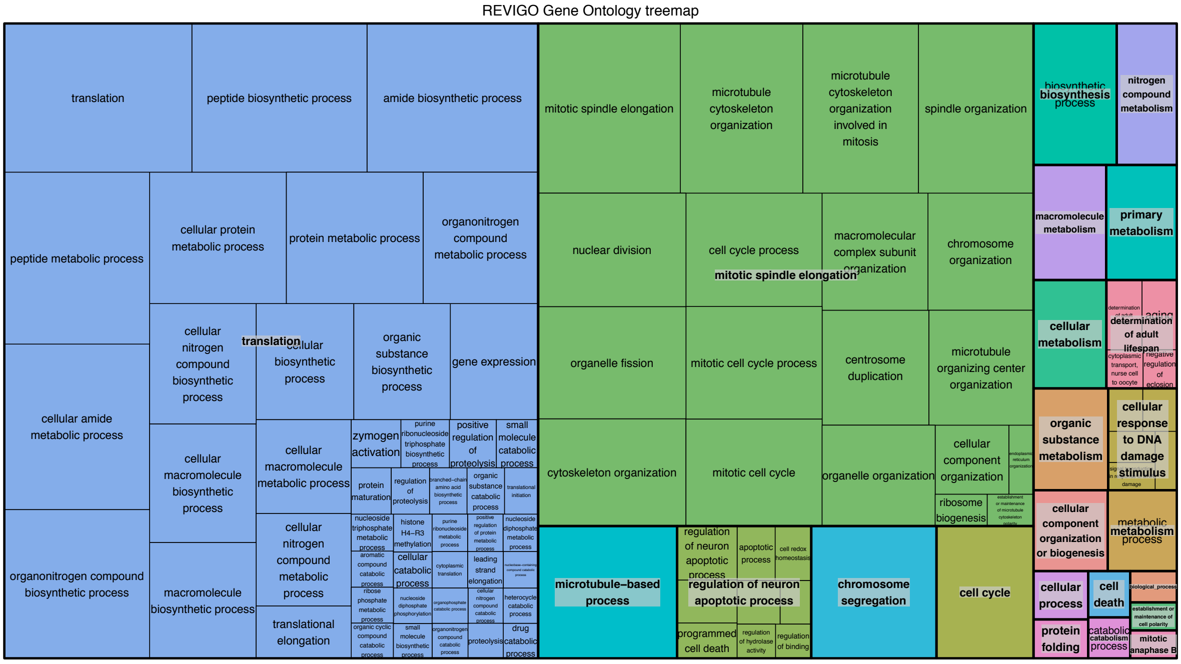

1.3 GO terms inferred from down-regulated DEGs 1 hour post-infection in *G. calmariensis*

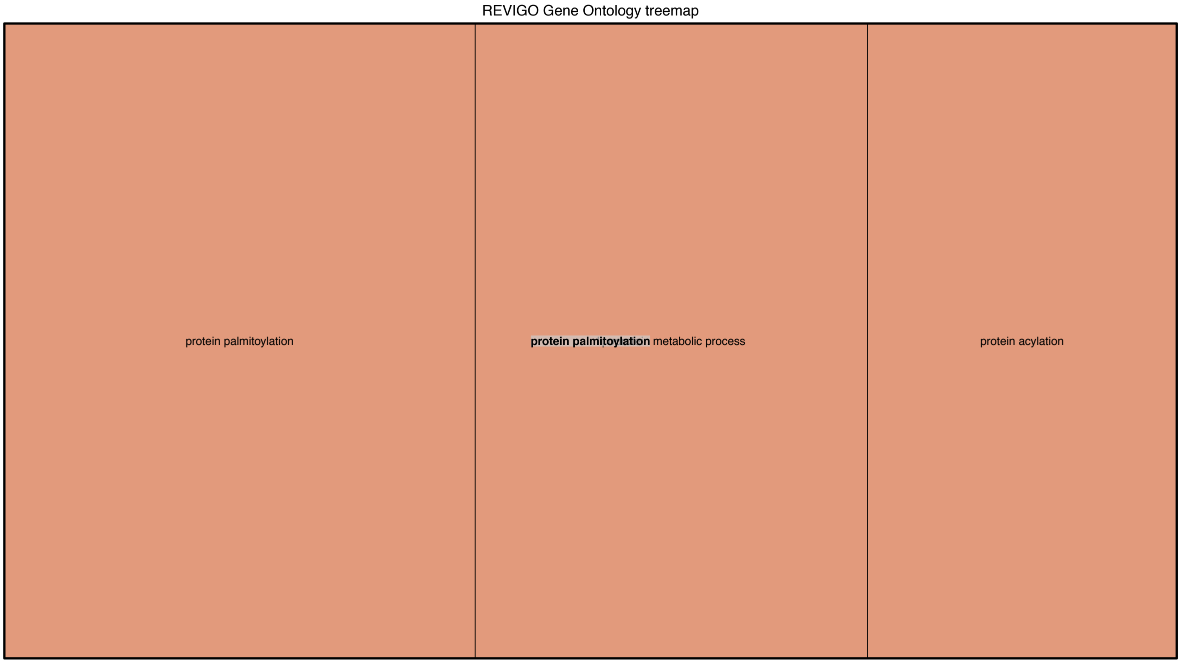

2.1 GO terms inferred from all DEGs 4 hour post-infection in *G. calmariensis*

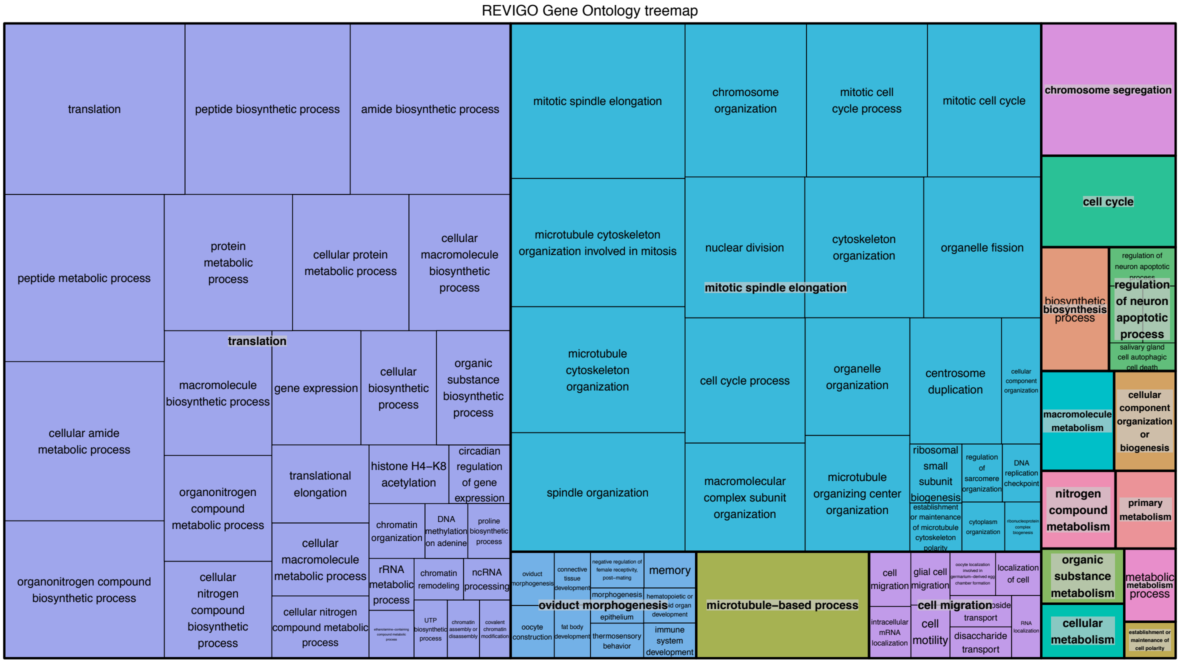

2.2 GO terms inferred from up DEGs 4 hour post-infection in *G. calmariensis*

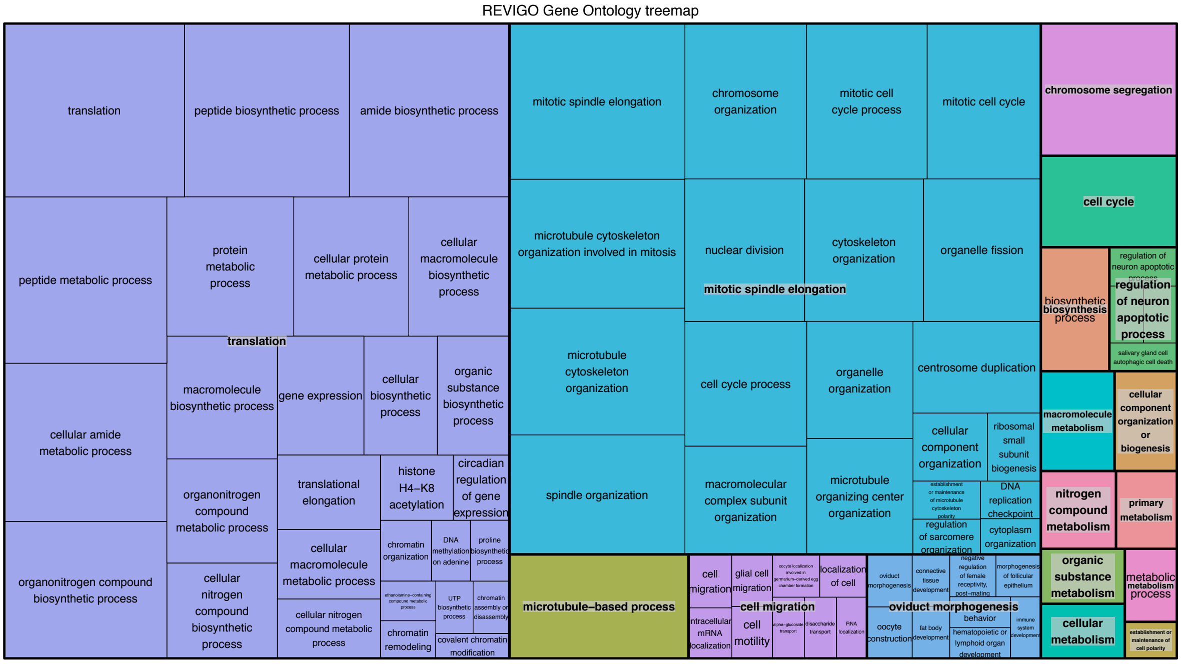

2.3 GO terms inferred from down DEGs 4 hour post-infection in *G. calmariensis*

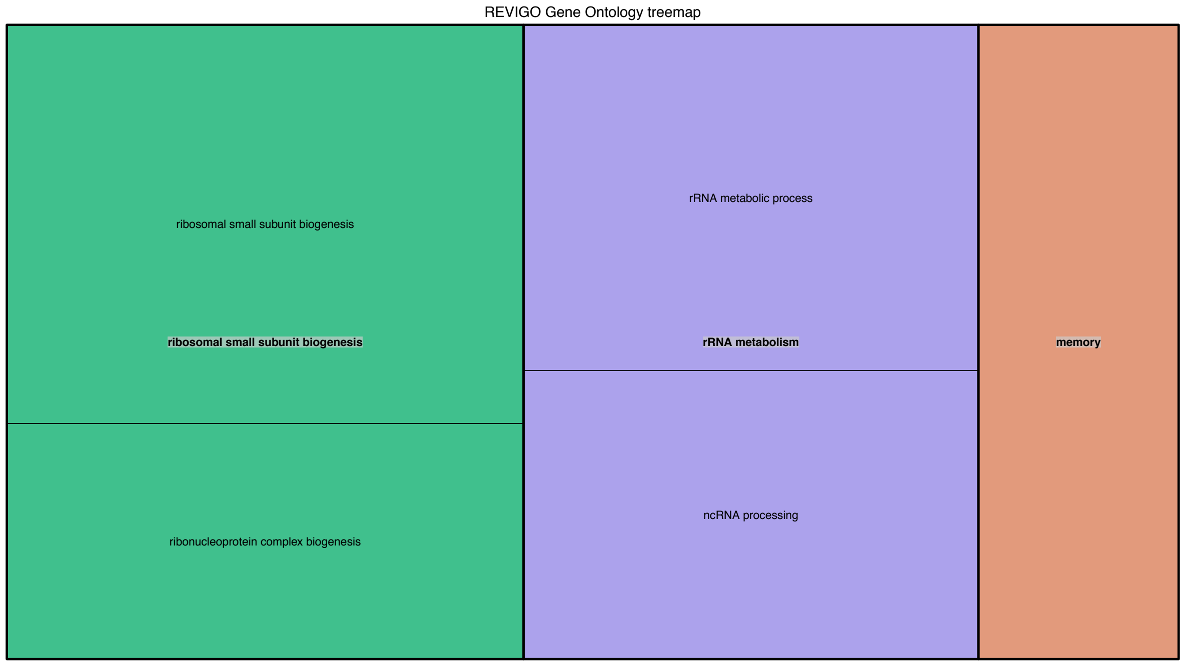

3.1 GO terms inferred from all DEGs 1 - 4 hours post-infection in *G. calmariensis*

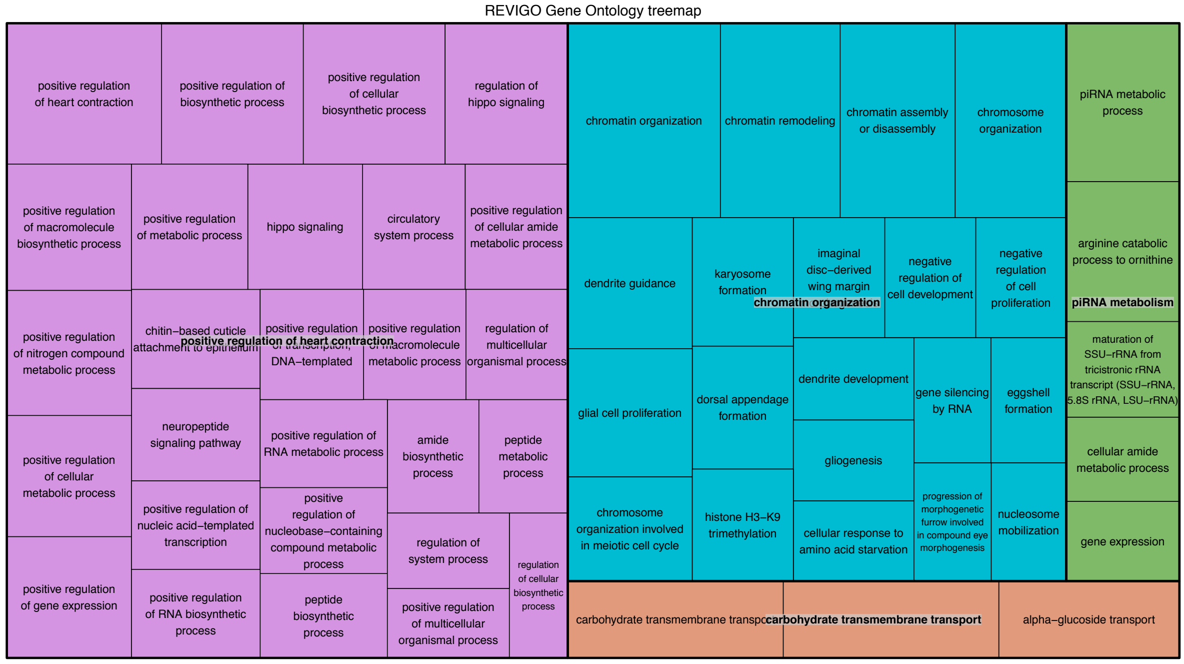

3.2 GO terms inferred from up-regulated DEGs 1 - 4 hours post-infection in *G. calmariensis*

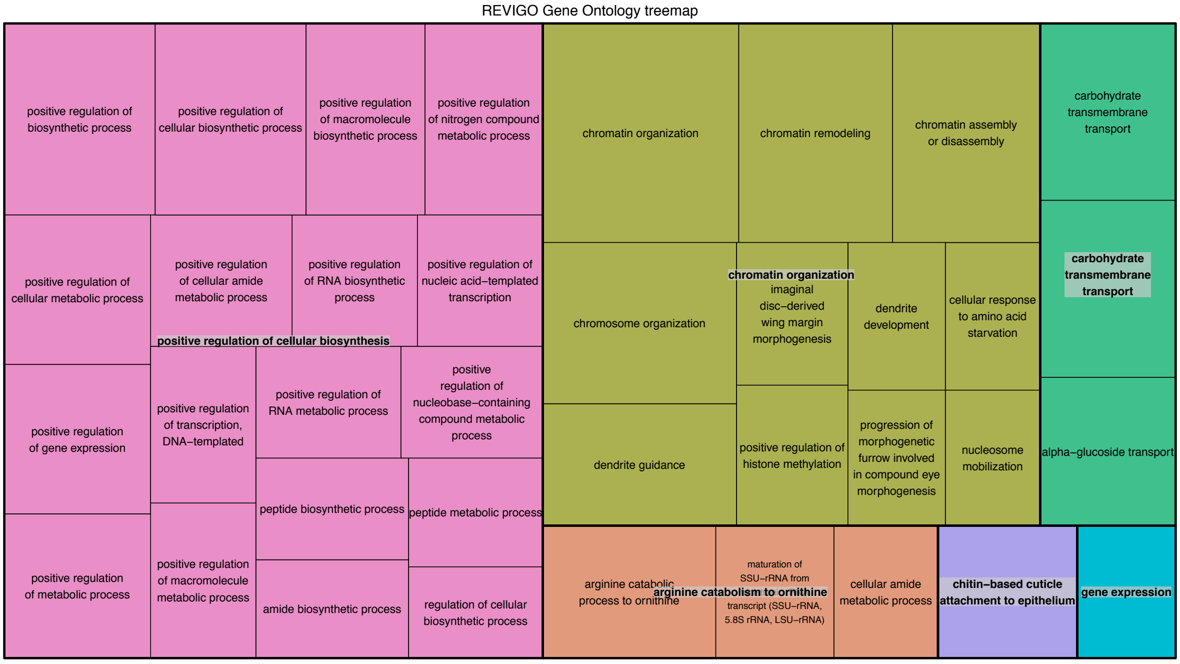

3.3 GO terms inferred from down-regulated DEGs 1 - 4 hours post-infection in *G. calmariensis*

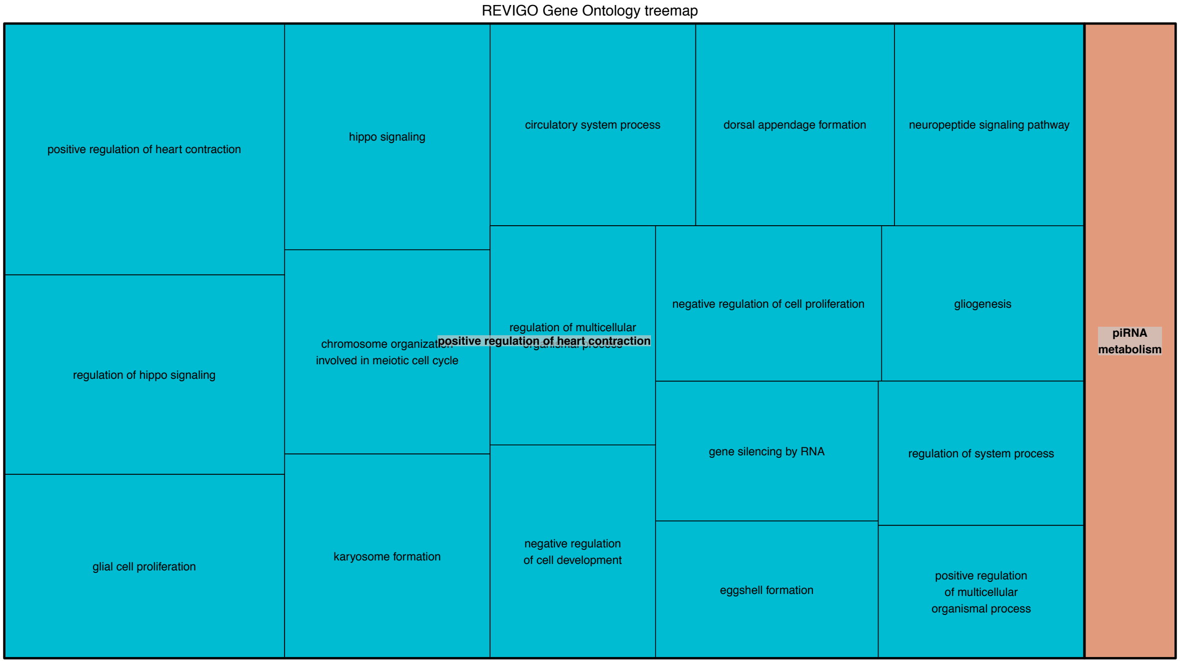

4.1 GO terms inferred from all DEGs 12 hour post-infection in *G. calmariensis*

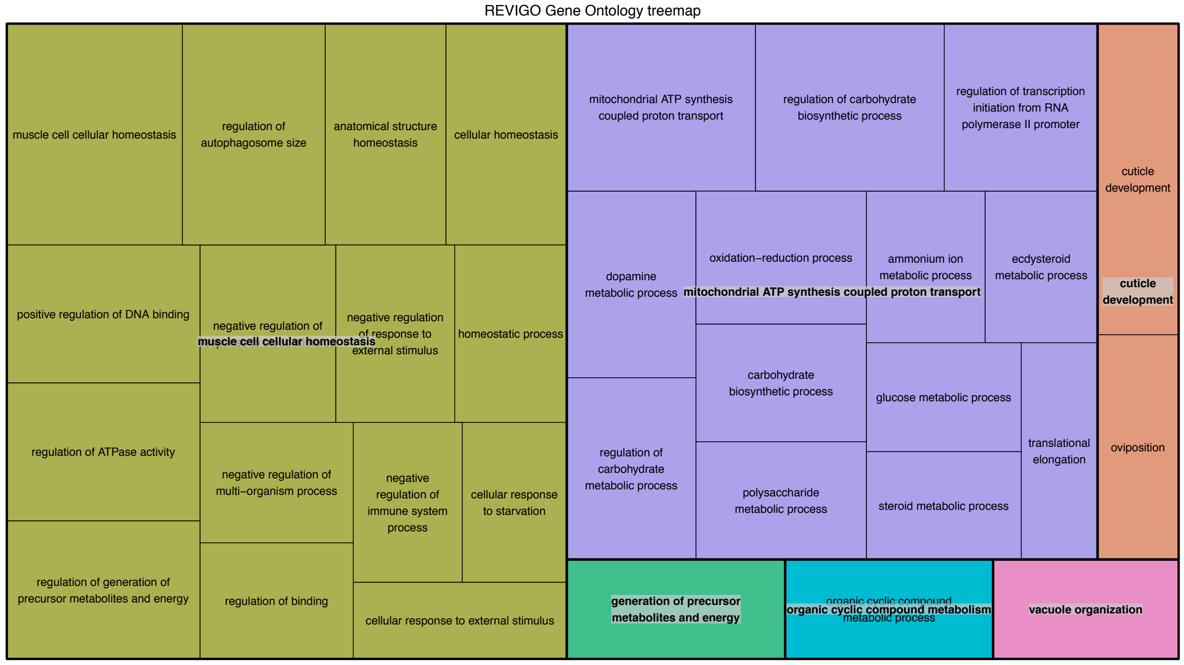

4.2 GO terms inferred from up-regulated DEGs 12 hour post-infection in *G. calmariensis*

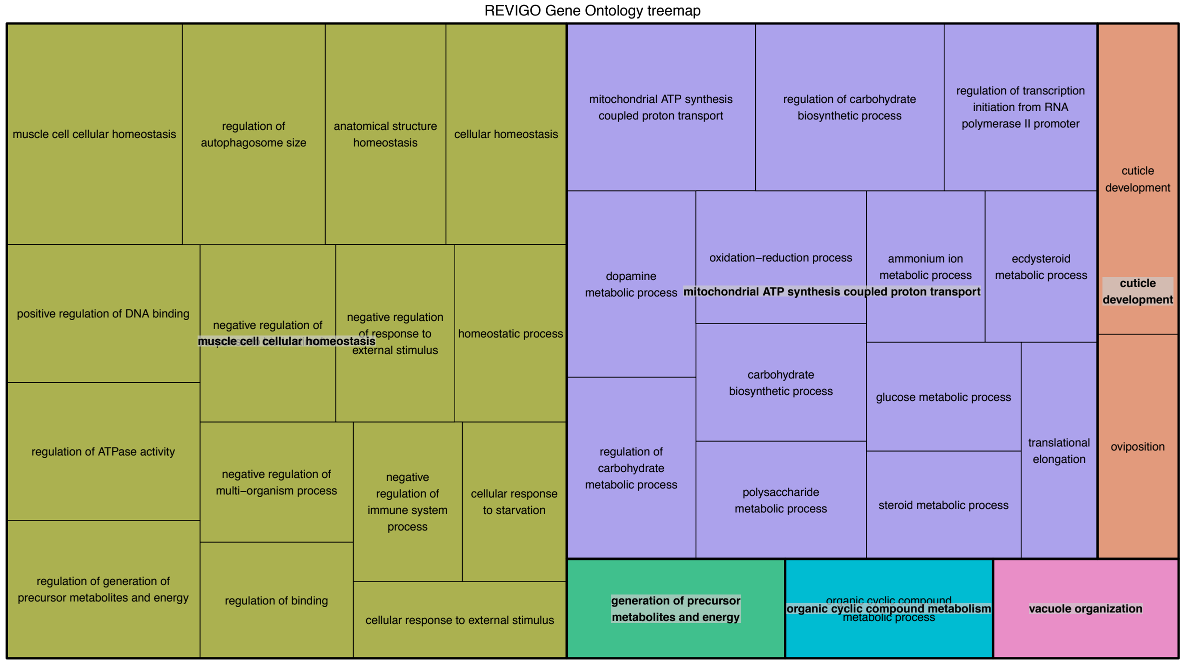

4.3 GO terms inferred from down-regulated DEGs 12 hour post-infection in *G. calmariensis*

No over-represented GO term was identified in this class.

5.1 GO terms inferred from all DEGs 1 hour post-infection in *G. pusilla*

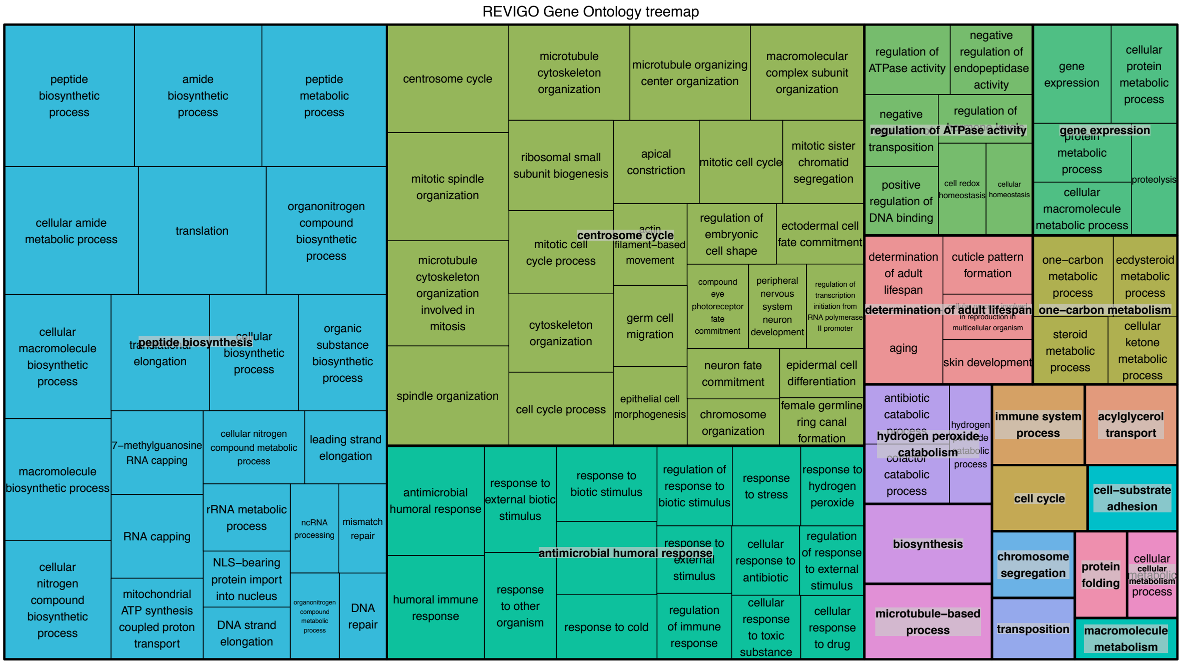

5.2 GO terms inferred from up-regulated DEGs 1 hour post-infection in *G. pusilla*

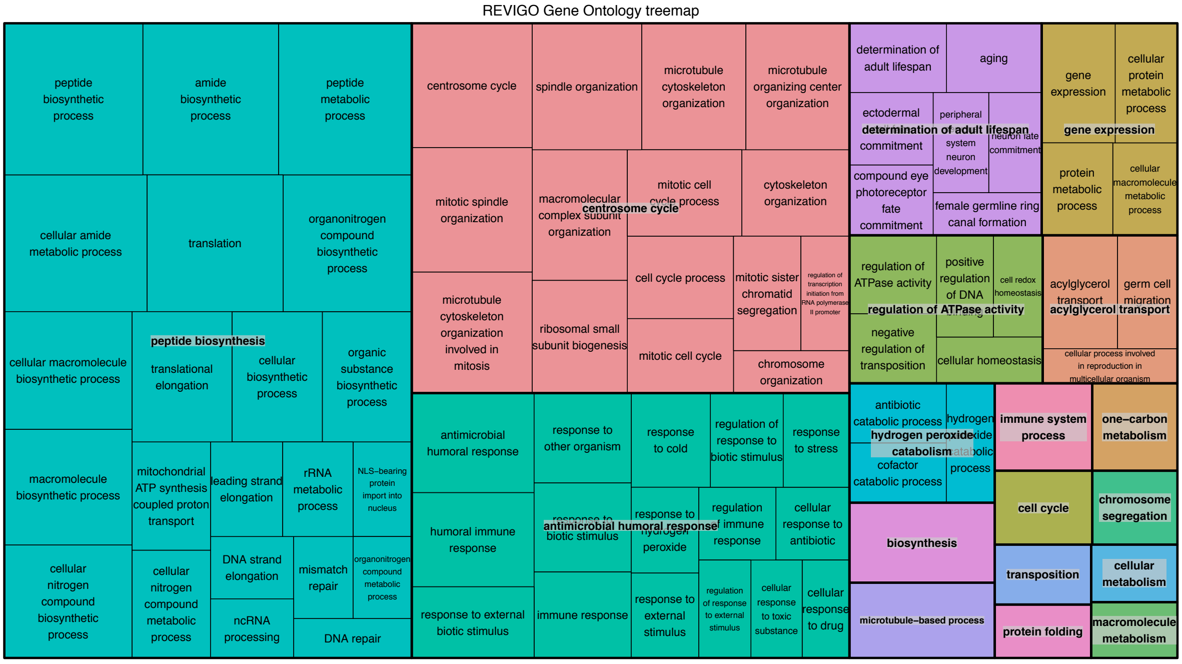

5.3 GO terms inferred from down-regulated DEGs 1 hour post-infection in *G. pusilla*

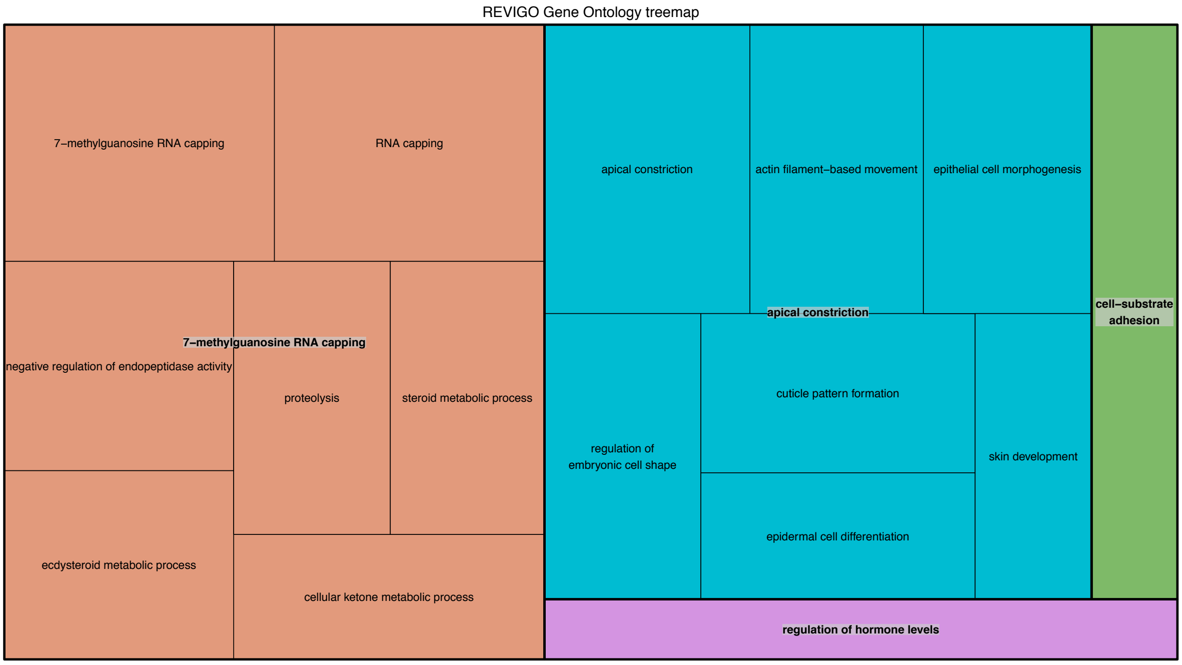

6.1 GO terms inferred from all DEGs 4 hour post-infection in *G. pusilla*

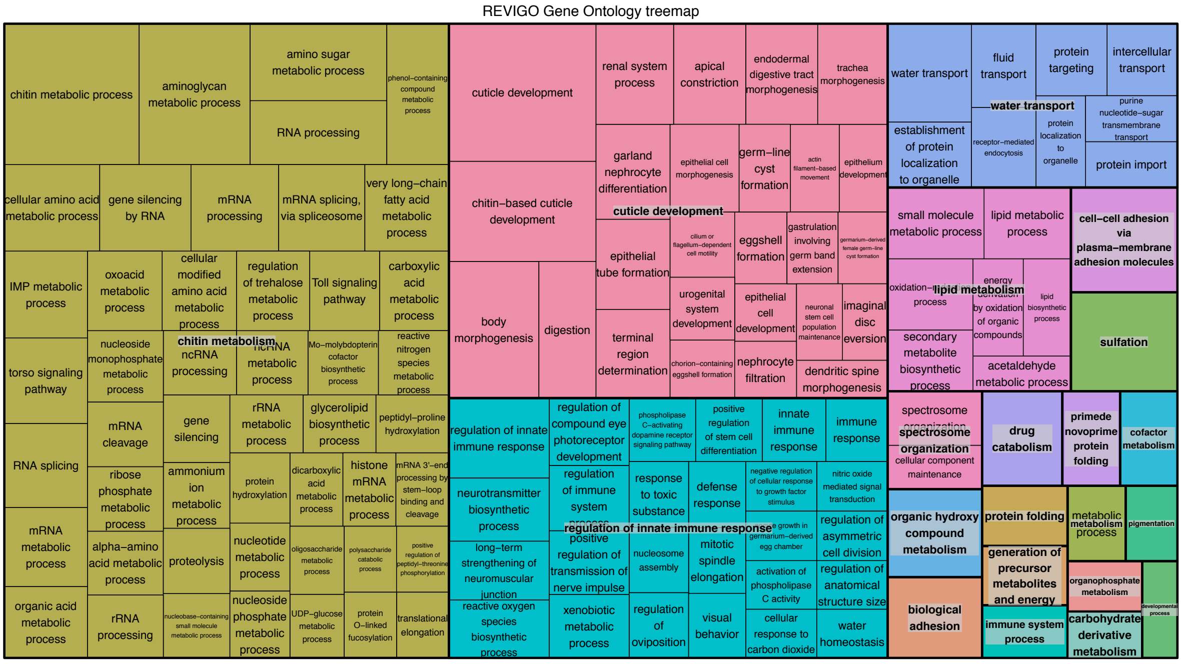

6.2 GO terms inferred from up-regulated DEGs 4 hour post-infection in *G. pusilla*

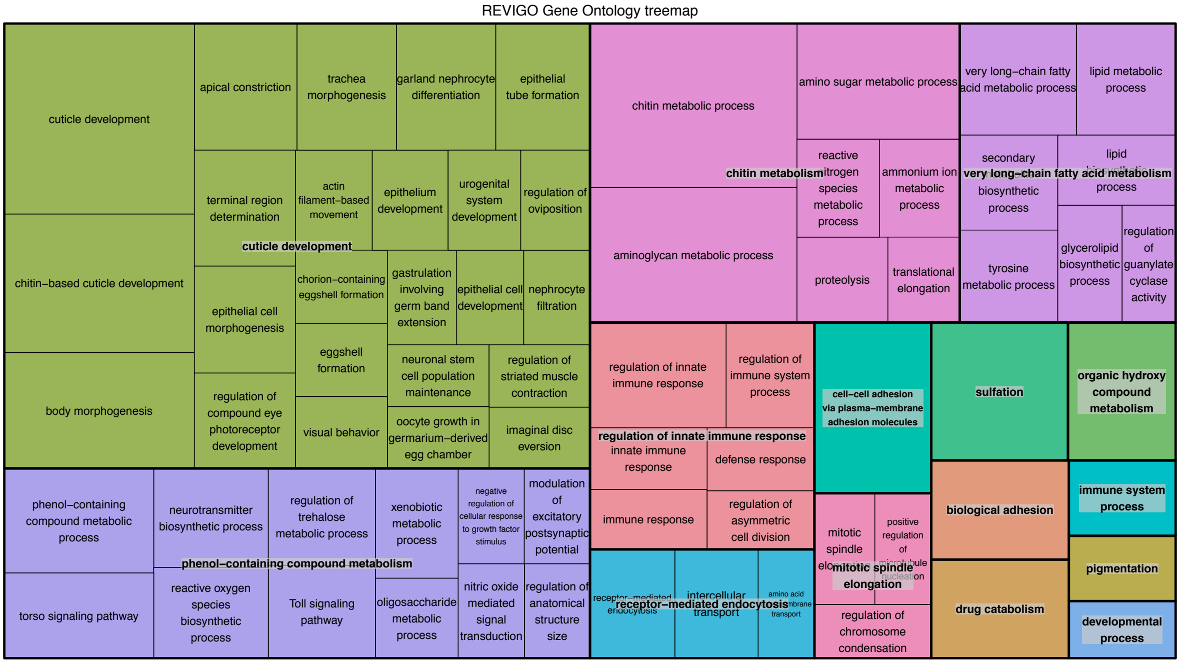

6.3 GO terms inferred from down-regulated DEGs 4 hour post-infection in *G. pusilla*

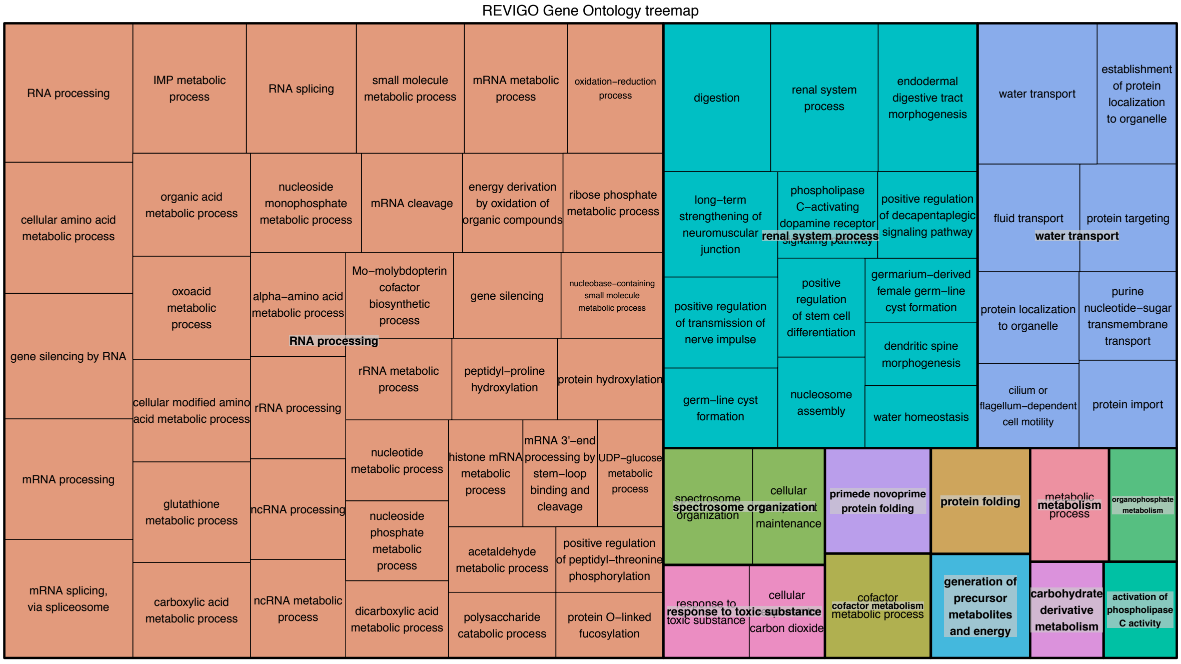

7.1 GO terms inferred from all DEGs 1 - 4 hours post-infection in *G. pusilla*

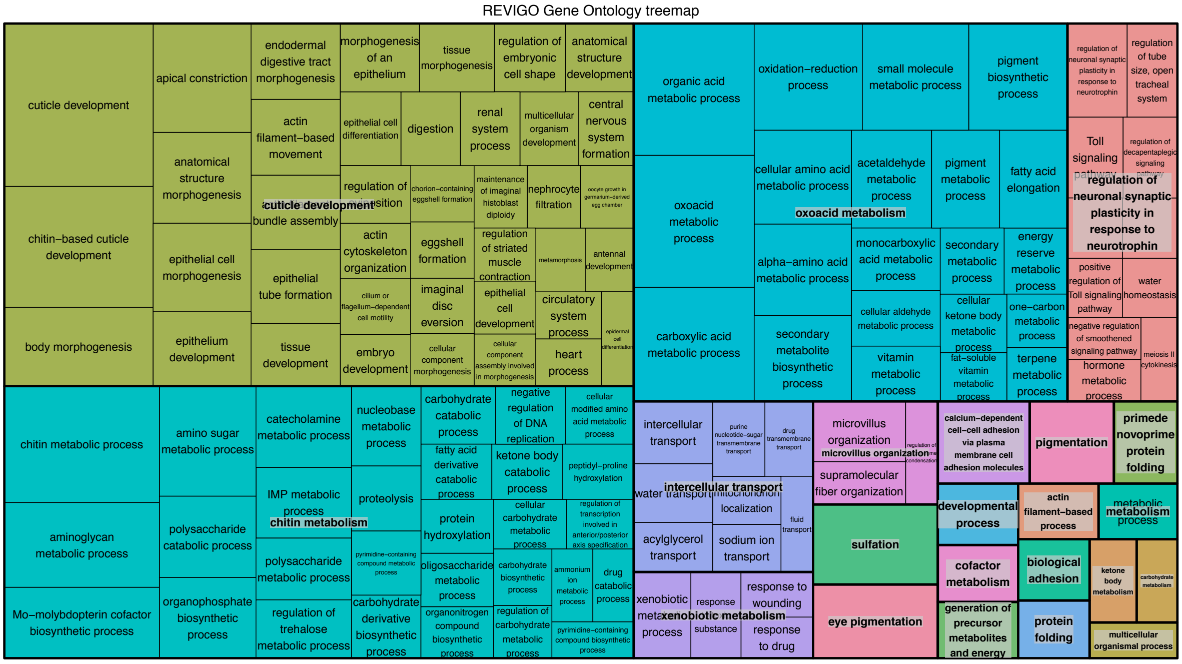

7.2 GO terms inferred from up-regulated DEGs 1 - 4 hours post-infection in *G. pusilla*

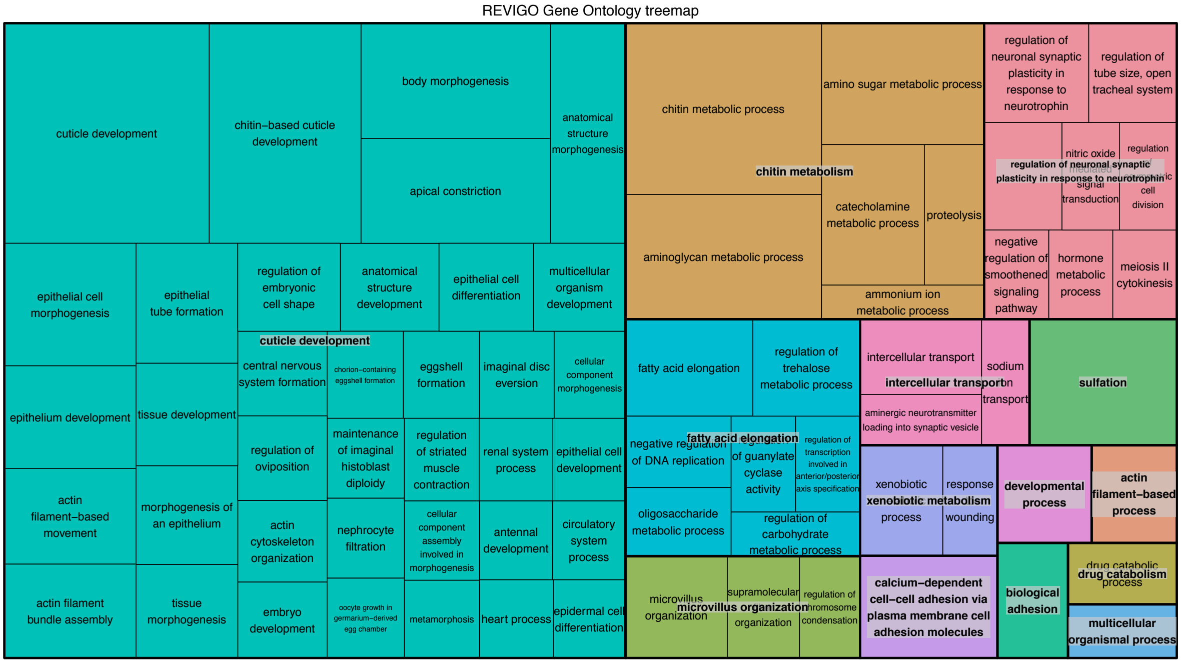

7.3 GO terms inferred from down-regulated DEGs 1 - 4 hours post-infection in *G. pusilla*

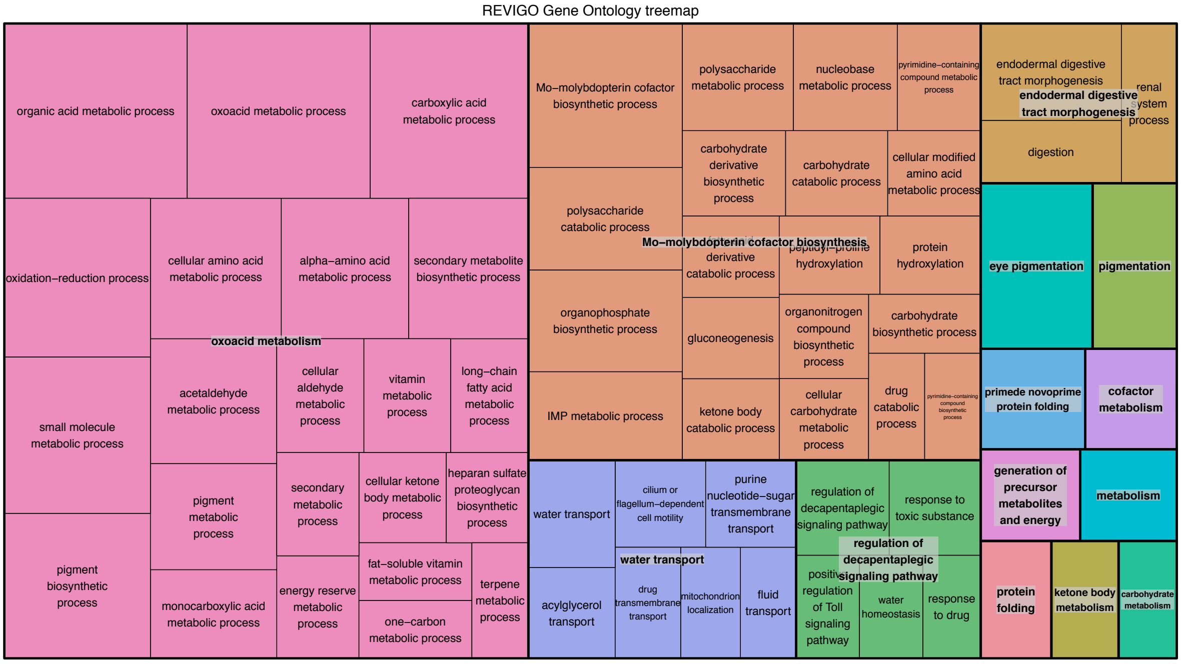

8.1 GO terms inferred from all DEGs 12 hour post-infection in *G. pusilla*

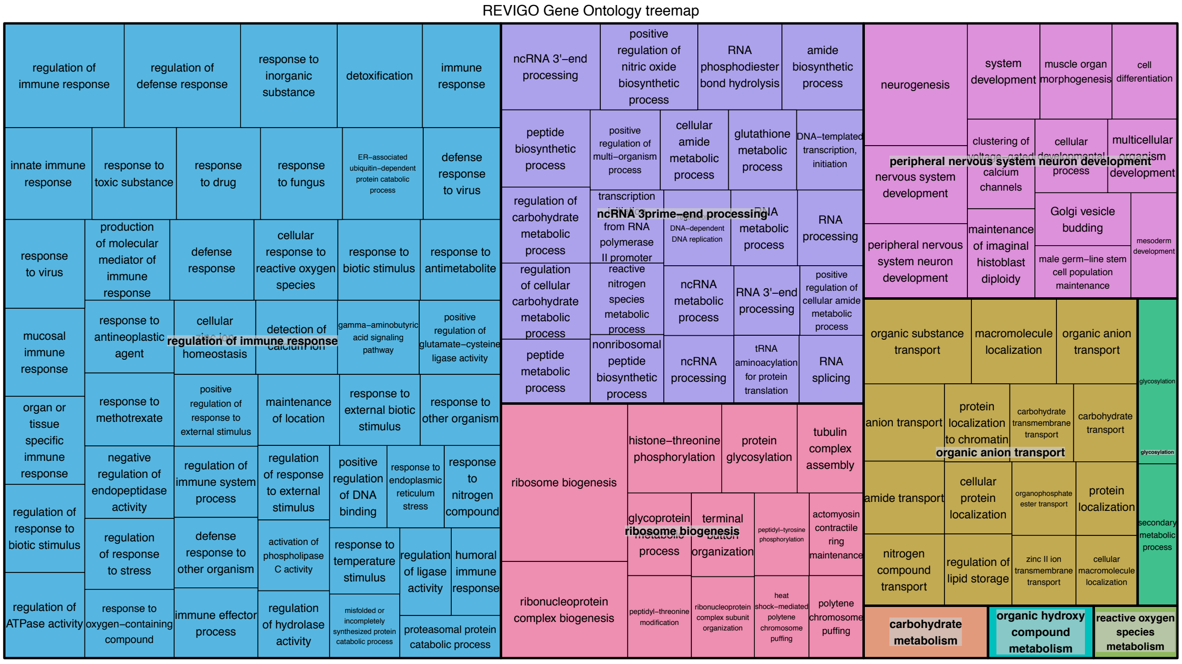

8.2 GO terms inferred from up-regulated DEGs 12 hour post-infection in *G. pusilla*

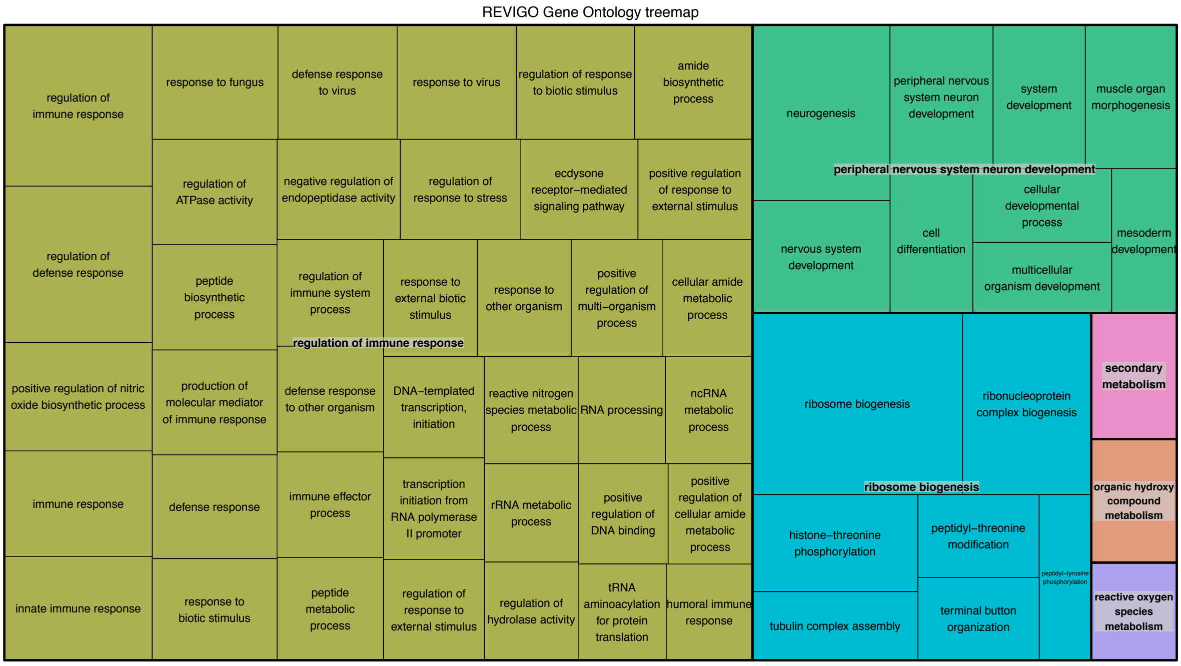

8.3 GO terms inferred from down-regulated DEGs 12 hour post-infection in *G. pusilla*

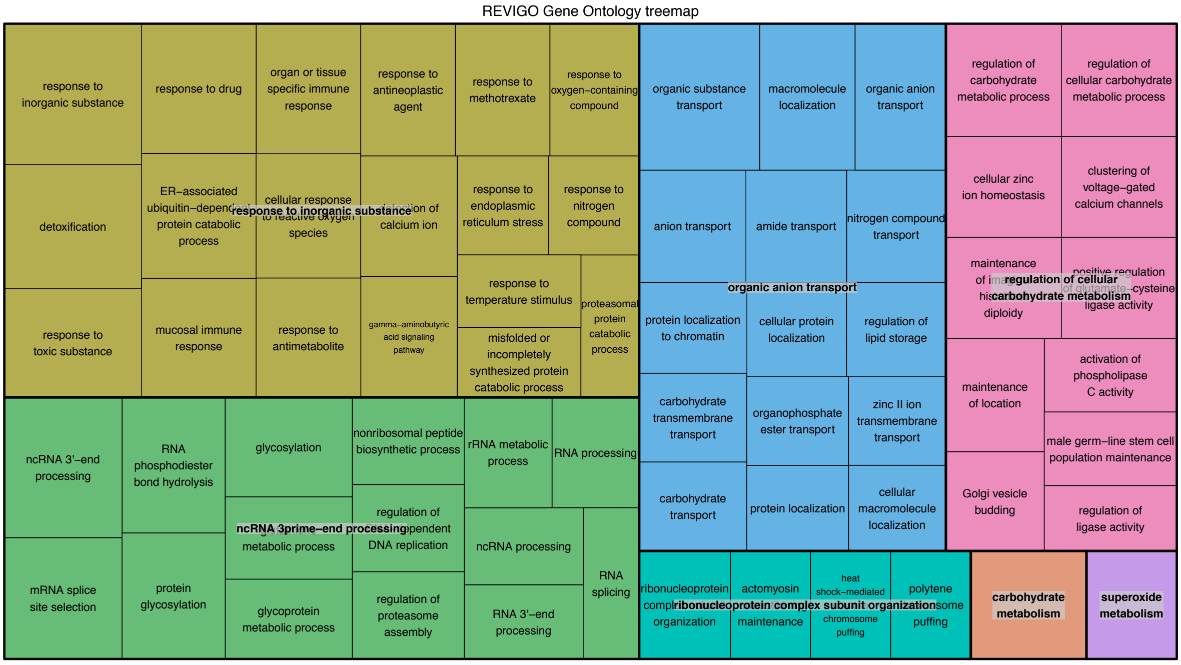

Supplement: evaa075_Supplementary_Data [file evaa075_supplementary_data.zip › Figure S2.pdf]
