# Supplementary material for: Differential Expression of Immune Genes between Two Closely Related Beetle Species with Different Immunocompetence following Attack by Asecodes parviclava
Source: Genome Biol Evol. 2020 Apr 13;12(5):522–34. doi: 10.1093/gbe/evaa075 (PMC7211424; doi:10.1093/gbe/evaa075)

Figure S3.

Schematic representation of immune pathways expressed under parasitoid attack four hours after parasitoid attack in signalling and recognition pathways in A) *G. pusilla* and B) *G. calmariensis*; Hematopoiesis process in C) *G. pusilla* and D) *G. calmariensis*. Dashed-line circles correspond to proteins known to be in the pathway in *Drosophila* but which were not detected in the *Galerucella* transcriptome. Purple circles represents genes that were upregulated in infected samples compared with non-infected ones. Some genes with obscure or unknown immune functions are not listed in the figure. Figure adapted from Wertheim et al [4].

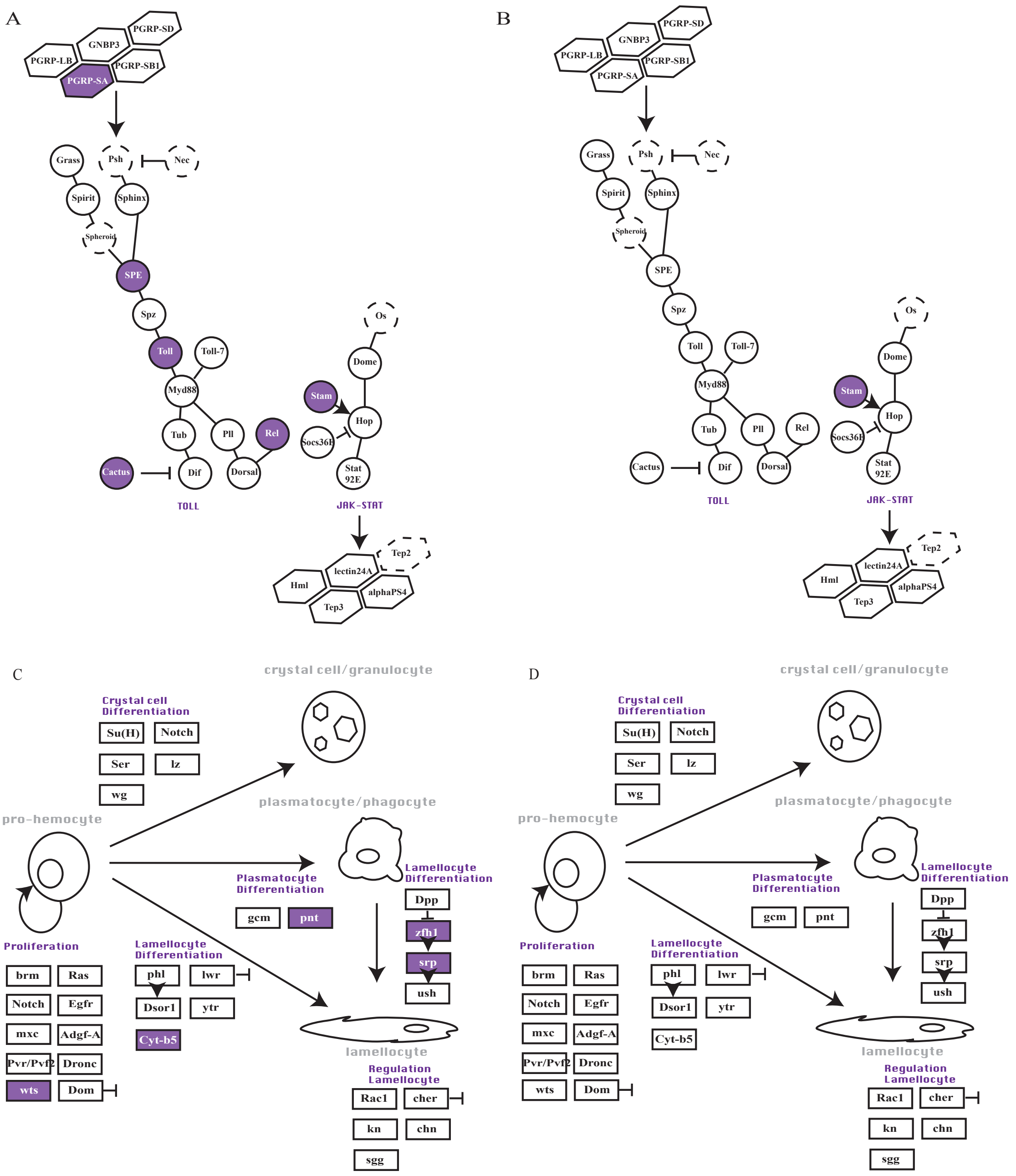

Supplement: evaa075_Supplementary_Data [file evaa075_supplementary_data.zip › Figure S3.pdf]
